# S100B inhibition protects from chronic experimental autoimmune encephalomyelitis

**DOI:** 10.1093/braincomms/fcac076

**Published:** 2022-03-25

**Authors:** Catarina Barros, Andreia Barateiro, Alexandre Neto, Beatriz Soromenho, Afonso P. Basto, Joana M. Mateus, Sara Xapelli, Ana M. Sebastião, Dora Brites, Luís Graça, Adelaide Fernandes

**Affiliations:** 1 Research Institute for Medicines (iMed.ULisboa), Faculdade de Farmácia, Universidade de Lisboa, 1600-083 Lisbon, Portugal; 2 Department of Pharmaceutical Sciences and Medicines, Faculdade de Farmácia, Universidade de Lisboa, 1600-083 Lisbon, Portugal; 3 Instituto de Medicina Molecular João Lobo Antunes, Faculdade de Medicina, Universidade de Lisboa, 1649-028 Lisbon, Portugal; 4 CIISA – Centro de Investigação Interdisciplinar em Sanidade Animal, Faculdade de Medicina Veterinária, Universidade de Lisboa, 1300-477 Lisbon, Portugal; 5 Instituto de Farmacologia e Neurociências, Faculdade de Medicina, Universidade de Lisboa, 1649-028 Lisbon, Portugal

**Keywords:** multiple sclerosis, experimental autoimmune encephalomyelitis, S100B, neuroinflammation, immunity

## Abstract

Studies have correlated excessive S100B, a small inflammatory molecule, with demyelination and associated inflammatory processes occurring in multiple sclerosis. The relevance of S100B in multiple sclerosis pathology brought an emerging curiosity highlighting its use as a potential therapeutic target to reduce damage during the multiple sclerosis course, namely during inflammatory relapses. We examined the relevance of S100B and further investigated the potential of S100B-neutralizing small-molecule pentamidine in chronic experimental autoimmune encephalomyelitis. S100B depletion had beneficial pathological outcomes and based on promising results of a variety of S100B blockade strategies in an *ex vivo* demyelinating model, we choose pentamidine to assay its role in the *in vivo* experimental autoimmune encephalomyelitis. We report that pentamidine prevents more aggressive clinical symptoms and improves recovery of chronic experimental autoimmune encephalomyelitis. Blockade of S100B by pentamidine protects against oligodendrogenesis impairment and neuroinflammation by reducing astrocyte reactivity and microglia pro-inflammatory phenotype. Pentamidine also increased regulatory T cell density in the spinal cord suggesting an additional immunomodulatory action. These results showed the relevance of S100B as a main driver of neuroinflammation in experimental autoimmune encephalomyelitis and identified an uncharacterized mode of action of pentamidine, strengthening the possibility to use this drug as an anti-inflammatory and remyelinating therapy for progressive multiple sclerosis.

## Teaser

Barros *et al.* report that S100B deletion is beneficial in an animal model of chronic multiple sclerosis, setting the rationale for S100B modulation by pentamidine, an S100B-binding drug, to ameliorate disease development and reduce inflammatory/immune response. These data strengthened the use of S100B inhibition for effective multiple sclerosis treatment.

## Graphical Abstract

**Graphical Abstract:**
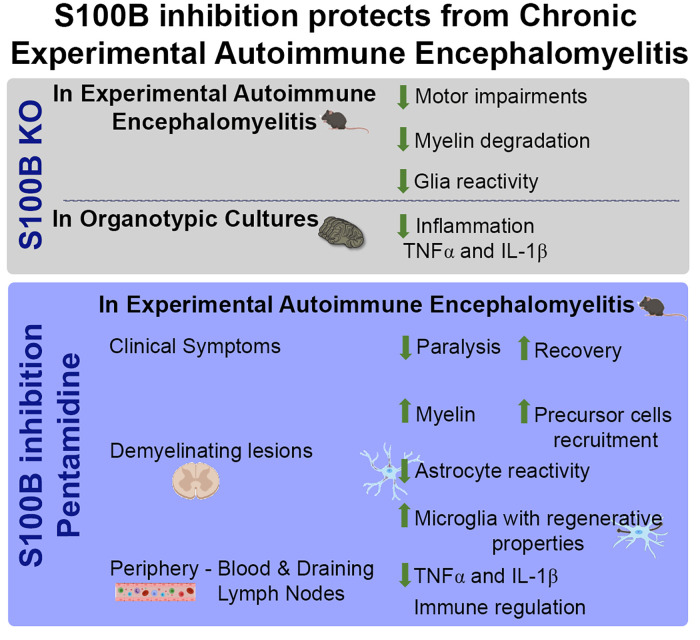

## Introduction

Multiple sclerosis is an autoimmune and chronic demyelinating disorder of the CNS that causes motor and cognitive disability among young and middle-aged adults.^1,2^ Pathologically, it is known that immune cells become overactivated against self-myelin proteins accompanied by increased activation of resident glial cells that exacerbate neuroinflammation.^3^

Inside the inflammatory environment, S100B, a small Ca^2+^ binding protein highly expressed by astrocytes, was described to be a potential biomarker of disease pathology.^4,5^ Despite its neurotrophic effects at nanomolar concentrations, S100B has detrimental consequences under pathological conditions, including glial activation, enhanced inflammation and oligodendrogenesis impairment that hampers possible remyelination.^6^ In addition, S100B may act as a damage-associated molecular pattern molecule (DAMP), or alarmin, in a variety of disorders being released to microenvironments to trigger tissue reaction.^7^ Particularly, in multiple sclerosis, S100B levels in both CSF and blood have been shown to reflect disease severity and progression.^4,5,8^ We recently showed a direct correlation between augmented S100B levels in human CSF and serum samples at the time of diagnosis of relapsing–remitting multiple sclerosis patients, and its high secretion by astrocytes in active and chronic-active lesions in post-mortem brain samples.^9^ Several attempts to target S100B toxic levels through antibody-mediated neutralization (e.g. anti-S100B, anti-S100 or anti-soluble S100B receptor, the receptor for advanced glycation end products, RAGE),^9–11^ specific S100B receptor antagonists (e.g. FPS-ZM1)^12^ and inhibitors of S100B synthesis (e.g. arundic acid)^13^ were already tested using *ex vivo* and *in vivo* models. Furthermore, the use of the S100B inhibitor pentamidine, an approved antiprotozoal drug, was shown to reduce S100B and RAGE expression in an Alzheimer’s disease animal model and elicit a neuroprotective role in hippocampal neurons.^14^ Recently, a study demonstrated that pentamidine partially ameliorates disease progression in the relapsing–remitting *in vivo* model of multiple sclerosis, the experimental autoimmune encephalomyelitis (EAE), by slightly improving EAE paralysis and cerebellar neuroinflammation, although its effect on spinal cord injury and CNS immunity was not explored.^15^

Here, we initially explored the effects of S100B deletion and then investigated the pharmacological value of pentamidine in the blockade of S100B toxic effects in chronic EAE, a preclinical mouse model of chronic multiple sclerosis. Induction of EAE in S100B knockout (KO) mice ameliorated clinical symptoms with reduced number of lesions and glial reactivity, corroborated by reduced inflammation in a demyelinating *ex vivo* model. Furthermore, treatment with pentamidine changed disease course and outcome, ameliorating disease severity and reducing locomotor impairment. Pathologically, pentamidine prevented lesion formation and oligodendrocyte impairment, in parallel with a decrease in astrocytic reactivity, and increased recruitment of non-pro-inflammatory microglia/macrophages cells possibly favouring remyelinating processes. In addition, pentamidine enhanced CNS infiltration by regulatory T (Treg) cells in opposite to T helper (Th)1 and Th17. Altogether, this study strengthened the crucial role of S100B in multiple sclerosis pathology and highlighted the possibility to use pentamidine as a therapy to reduce damage and improve recovery of multiple sclerosis lesions, with high drug repurposing potential.

## Materials and methods

### Animals

S100B KO mice progenitors were purchased from the Jackson Laboratory. The colonies were established in Instituto de Medicina Molecular João Lobo Antunes (Lisbon, Portugal), and the genotyping was done by polymerase chain reaction (PCR), in which three pairs of primers were used to amplify different DNA regions according to the manufacturer’s instructions (Wellcome Trust Sanger Institute, Hinxton Cambridge, UK). Female C57BL/6 mice were acquired from Instituto Gulbenkian Ciência (Oeiras, Portugal) and maintained at Instituto de Medicina Molecular João Lobo Antunes (Lisbon, Portugal). All animals were maintained under specific pathogen-free housing conditions, on a reverse 12h–12 h light/dark cycle and fed with regular chow and water *ad libitum*. Training and testing began between 8 and 11 weeks of age, after 1 week of acclimatization to the housing conditions.

Animal care followed the recommendation of the European Convention for the Protection of Vertebrate Animals Used for Experimental and Other Scientific Purposes (Council Directive 86/609/EEC) and National Law 1005/92 (rules for the protection of experimental animals). All animal procedures were approved by the Institutional Animal Care and Use Committee, and the national animal affairs regulatory office (Direção Geral de Alimentação e Veterinária). Every effort was made to minimize the number of animals used and their suffering.

### *Ex vivo* model of demyelination

Organotypic cerebellar slice cultures (OCSCs) treated with lysophosphatidylcholine (LPC) were prepared and analysed as described.^10^ Cerebella were obtained from S100B WT and S100B KO mice at postnatal Day 10 and cut into parasagittal slices with 400-μm thickness using a McIlwain tissue chopper. Four slices from different animals were placed into a 0.4-µm pore membrane culture insert (BD Falcon) in 6-well cell culture plates, in an air–liquid interface, at 37°C and 5% CO_2_ conditioned atmosphere and kept in culture until 7 days *in vitro* (DIV) to allow the clearance of debris and full myelination. For the first 3 days, slices were cultured with a culture medium consisting of 50% minimal essential media (Gibco, Life Technologies), 25% of both heat-inactivated horse serum (Gibco) and Earle’s balanced salt solution (Gibco), 6.5 mg/ml glucose (Gibco), 36 mM 4-(2-hydroxyethyl)-1-piperazineethanesulfonic acid (HEPES) (Biochrom AG) and 1% of both l-glutamine (Sigma-Aldrich) and antibiotic–antimycotic (Sigma-Aldrich). The culture media was renewed every day. At 4 DIV, to improve neuronal viability, culture media was replaced by a serum-free media, containing Neurobasal-A (NB, Gibco), supplemented with 2% B-27 (Gibco), 1% l-glutamine, 36 mM glucose, 1% of antibiotic–antimycotic and 25 mM HEPES. Again, the media was daily replaced. After 7 DIV, slices were incubated with 0.5 mg/ml LPC in serum-free culture media for 18 h, at 37°C, after which the medium was changed into serum-free media. Cultures were maintained for 30 h and then slices were collected and stored for RNA extraction or fixed for immunohistochemistry assays.

### Experimental autoimmune encephalomyelitis induction

Female C57BL/6 mice with 11 weeks were EAE-induced using a commercial kit (Hooke Laboratories), according to the manufacturer’s instructions. Animals were subcutaneously injected in the upper and lower back with a total of 0.2 ml (100 µl per site) of the myelin oligodendrocyte glycoprotein (MOG) 35–55 peptides emulsified in complete Freund’s adjuvant (CFA). In addition, to achieve full immunization, animals were administered intraperitoneally with a total of 100 ng (100 µl per animal) of pertussis toxin (PTx) in PBS on the first day of immunization and repeated on the following day (1-day post-EAE induction, dpi). During the EAE procedure, the mice were not submitted to anaesthesia, and all the procedures were performed by the same person among all experimental groups.

After EAE induction, all animals were weighed and monitored daily. Clinical signs of EAE were also assessed using a scale ranging from 0 to 5 grades that are, briefly, characterized by an ascending paralysis beginning at the tail (Score 1), followed by limb and forelimb paralysis (Score 2 and 3, respectively), leading to quadriplegia (Score 4) and complete paralysis (Score 5). Paralyzed animals were granted easier access to food and water. Importantly, the EAE model was generally considered a success if it exceeds a score of 2. Moreover, animals that had to be sacrificed taking into account humane end-point, were given the last clinical evaluation for the rest of the experiment.

### Experimental groups and pentamidine treatment

For initial studies, female S100B KO and S100B WT mice with the same genetic background were EAE-induced, with five to eight animals per group. For pharmacological studies, female C57BL/6 mice were randomly divided into three different experimental groups in two independent experiments, 5–15 animals per group. Non-induced animals (or naïve group) were injected subcutaneously with an emulsion of PBS in CFA and were injected with PTx at the same concentration and timepoints, as previously described. The EAE-induced animals (or EAE group) were treated daily with vehicle solution, PBS, as well as the naïve group. Pentamidine, an antiprotozoal drug, was obtained from Sigma-Aldrich. Pentamidine treatment (or EAE + Pnt group, 4 mg/kg of body weight, based on studies for acute colitis^16^) was given daily by intraperitoneal injection, one time per day, beginning on the day of disease induction for prophylactic studies and at the first day of clinical symptoms for individual mice (clinical score equal to 0.5 or 1) for therapeutic studies. EAE groups were sacrificed at 17 and 30 dpi together with the naïve group.

### Motor coordination testing

Mouse motor assessment by pole test and rotarod protocols was performed and registered at 8, 11 and 14 dpi to assess disease progression. The animals were placed in a behaviour room and habituated for 30 min under dim yellow light before the tests. To remove any olfactory clues, the apparatuses were cleaned with a 30% ethanol solution between each animal for all tests. The experimenter remained in the room while the animals were being tested.

For the pole test, the system was composed of a square base with a rough-surface pole (height 50 cm; diameter 2 cm) fixed on a square wood base. The apparatus was placed on a cage covered with animals’ bedding material and paper rolls. Four trials with a minimum of 15 min interval between each were done. Mice were placed facing up on top of the pole and the time the animals took to turn down and reach the cage was evaluated. If the animal fell or did turn down and up again, this was considered a failed trial. To register the tests, the animals were videotaped for further analyses blinded to the evaluator.

Regarding the rotarod test, the mice were tested in a rotarod apparatus (Panlab, Harvard Apparatus, Barcelona, Spain) specific for mice to evaluate their motor performance. In the first day, animals experienced an habituation period, where they were placed on the rotating rod at 7 rotations per minute (rpm). This habituation period was considered over when the animals were able to stay on the rod without falling for a minimum of 2 min. Then, the animals were subjected to 3 trials on an accelerating protocol (a gradual increase from 4 to 40 rpm, during 5 min) with a minimum of 30 min between each trial. The latency to fall and respective rotation was registered automatically by the apparatus.

### Preparation of mononuclear cells and flow cytometry

Experimental groups were sacrificed at disease peak, 17 dpi. Animals were anaesthetized with a non-lethal dose of isoflurane and intracardially perfused with cold PBS. The spinal cord was mechanically dissociated and digested with collagenase Type VIII (0.2 mg/ml; Sigma-Aldrich) in PBS at 37°C for 30 min. To isolate mononuclear cells, tissue passed through a 70-µm strainer, followed by a 30% Percoll gradient and centrifugation for 20 min at 2000 rpm. Cells were recovered, resuspended in a complete medium and used for intracellular staining.

For staining, cells were stimulated for 4 h in a complete culture medium containing 50 ng/ml PMA, 500 ng/ml ionomycin, 10 µg/ml brefeldin A and Golgi Stop (Sigma-Aldrich). Staining with LIVE/DEAD Fixable Dead Cell Stain kit (Life Technologies) was performed before fixation to allow gating on viable cells. Cells were blocked for 20 min and stained for antibodies targeting specific surface markers for different lymphocytes (CD19-PerCPCy5.5, CD4-PECY7, CD25-APCeF780, CD8-BV421, CD44-BV605; all from eBioscience). After staining of surface markers, cells were treated with fixation and permeabilization kit (eBioscience) and stained with interferon (IFN)-γ-fluorescein isothiocyanate (FITC) (XMG1.2), interleukin (IL)-17A-PE (ebio 17B7) and Foxp3-APC (FJK165) antibodies from BD Biosciences and eBioscience according to the manufacturers’ recommendations.

### Mononuclear cells cytokine production assay

Draining lymph nodes were removed and red blood cells were eliminated using the ACK method. Cell suspensions were prepared and plated in 96-well flat-bottom plates in complete medium [RPMI 1640 with GlutaMAX, supplement with 10% fetal bovine serum (FBS), 1% HEPES, 1% penicillin/streptomycin, 1% sodium pyruvate, 0.1% 2-ME; all Invitrogen]. The culture was stimulated with 20 µg/ml of MOG_35–55_, at 37°C. After 72 h, the supernatants were recovered for quantification by enzyme linked immunosorbent assay (ELISA) Kits (Invitrogen) of IFN-γ, IL-17A and IL-10.

### Immunohistochemistry and data analysis

The lumbar spinal cord was post-fixed in 4% paraformaldehyde and then transferred to 30% sucrose solution. Then, the tissue was embedded in Tissue-Tek O.C.T compound (Sakura Finetek) and cross-sectioned in serial coronal cryostat sections with 20-µm thickness (Cryostat Leica CM S3050). Frozen sections were defrosted at room temperature (RT). After fixation, the tissue was permeabilized with 0.25% Triton X-100 in PBS for 10 min and then incubated for 1 h with blocking solution [5% bovine serum albumin (Sigma-Aldrich), 5% FBS and 0.1% Triton X-100 (Roche Diagnostics) in PBS]. Then, the sections were incubated with primary antibodies diluted in a blocking solution for 48 h at 4°C. The following antibodies were used: myelin basic protein (MBP, 1:200, BioRad) for mature oligodendrocytes, neuron-glial antigen 2 (NG2, 1:100, Millipore) for oligodendrocyte precursor cells, glial fibrillary acidic protein (GFAP, 1:100, Novocastra) for astrocytes, ionized calcium-binding adapter molecule 1 (Iba1, 1:250, WAKO), inducible nitric oxide synthase (iNOS, 1:100, BD Biosciences), CX3 chemokine receptor 1 (CX3CR1, 1:200, Abcam), as well as S100B (1:250, DAKO). Following incubation, slices were washed three times for 10 min with PBS before incubation for 2 h at RT with the following secondary antibodies: anti-rabbit Alexa Fluor 488, anti-rat Alexa Fluor 594, anti-mouse Alexa Fluor 488 and 647 (1:500, Invitrogen, in blocking solution). Slices were washed three times for 10 min each, incubated for 5 min with DAPI (1:1000), washed three times for 5 min each with PBS and mounted with Fluoromount-G (Southern Biotech). Fluorescent images were obtained by confocal microscopy using Leica DMi8-CS inverted microscope with Leica LasX software (Leica Application Suite X), with 20× and 40× magnification. Approximately, 18–20 z-stacks were taken per slice per condition, reducing variation in image acquisition, and all analyses were done in the merged z-stacks. Demyelination and inflammation were analysed in white and grey matter. Furthermore, the percentage of area occupied by NG2, GFAP, S100B, iNOS, CX3CR1 and Iba1 in the absence of MBP was measured using Fiji software in each section. To correlate the glial activation, cell infiltration and the recruitment of oligodendrocyte precursor cells with white matter lesions, the areas of lesion plaque (P), periplaque (PP) and normal-appearing white matter (NAWM) were delimitated. The lesion plaque was identified by the loss of MBP staining and increase in cell nuclei with DAPI staining. The immediately adjacent area was defined as PP, selecting the 100 µm radius surrounding the plaque, and also a 100 µm adjacent radius to this PP area was defined as the NAWM. Results are given by averaging values determined in three different slices from each of five mice per group.

### Semi-quantitative qReal-Time PCR

Total RNA was extracted from the thoracic spinal cord and cerebellar slices from each experimental group, using RiboZol™ reagent method, according to the manufacturer’s guidelines (VWR Life Science). Total RNA was quantified using NanoDrop ND-100 Spectrophotometer (NanoDrop Technologies) and reversibly transcribed into complementary DNA (cDNA) with the Xpert cDNA Synthesis Mastermix Kit (GRiSP), under recommended conditions. Quantitative Real-Time PCR (qReal-Time PCR) was performed using β-actin as an endogenous control to normalize the expression level of: *S100B*, F- 5′ TGTAGACCCTAACCCGGAGG 3′ and R- 5′ TGCATGGATGAGGAAGGCAT 3′; tumour necrosis factor (TNF)α, F- 5′ TACTGAACTTCGGGGTGATTGGTCC 3′ and R- 5′ CAGCCTTGTCCCTTGAAGAGAACC 3′, *IL-1β*, F- 5′ CAGGCTCCGAGATGAACAAC 3′ and R- 5′ GGTGGAGAGCTTTCAGCTCATA 3′ and *IL-10*, F- 5′ ATGCTGCCTGCTCTTACTGA 3′ and R- 5′ GCAGCTCTAGGAGCATGTGG 3′. cDNA samples were amplified by qRT-PCR on a 7300 Real-Time PCR System (Applied Biosystem) by the excitation and emission of Xpert Fast SYBR MasterMix (GRiSP). The PCR was performed in 384-well plates, in which each sample was in duplicate and a no-template control was included. The cycle conditions were previously optimized: 50°C for 2 min, 95°C for 10 min followed by 40 cycles at 95°C for 15 s and 64°C for 1 min. To verify the specificity of the amplification, a melt-curve was performed immediately after the amplification protocol. Non-specific products of PCR were found in any case. Relative messenger RNA (mRNA) concentrations were calculated using the Pfaffl modification of the ΔΔCt equation cycle number at which fluorescence passes the threshold level of detection (Ct) Δ, taking into account the efficiency values of individual genes. The results were normalized to the endogenous gene, β-actin, and were obtained by the formula 2^−ΔΔCt^. ΔCt is the value obtained, for each sample, by performing the difference between the mean Ct value of each gene of interest and the mean Ct value of *β-actin*. ΔΔCt of one sample is the difference between its ΔCt value and the ΔCt of the sample chosen as reference.

### ELISA

Mouse TNFα, IL-1β, IFN-γ, IL-17A and IL-10 were measured in the serum and in the supernatant of non-stimulated/stimulated mononuclear cells isolated from draining lymph nodes by ELISA, following manufacturers protocol (Invitrogen).

### Statistical analysis

All results are presented as mean ± SEM. Data analysis was performed using PRISM GraphPad 7.0 (GraphPad Software, San Diego, CA, USA). Significant differences between the two groups were determined by unpaired two-tailed Student’s *t*-test; otherwise, Mann–Whitney U-test was applied. To assess significant differences between more than two groups and between parameters, one-way and two-way ANOVA with Tukey post-test for multiple comparisons were performed. Statistical significance was ranked *P < 0.05, **P < 0.01 and ***P < 0.001.

### Data availability

The authors confirm that the data supporting the findings of this study are available within the article and its supplementary material.

## Results

### S100B depletion partially protects from EAE-associated paralysis

**Figure 1:**
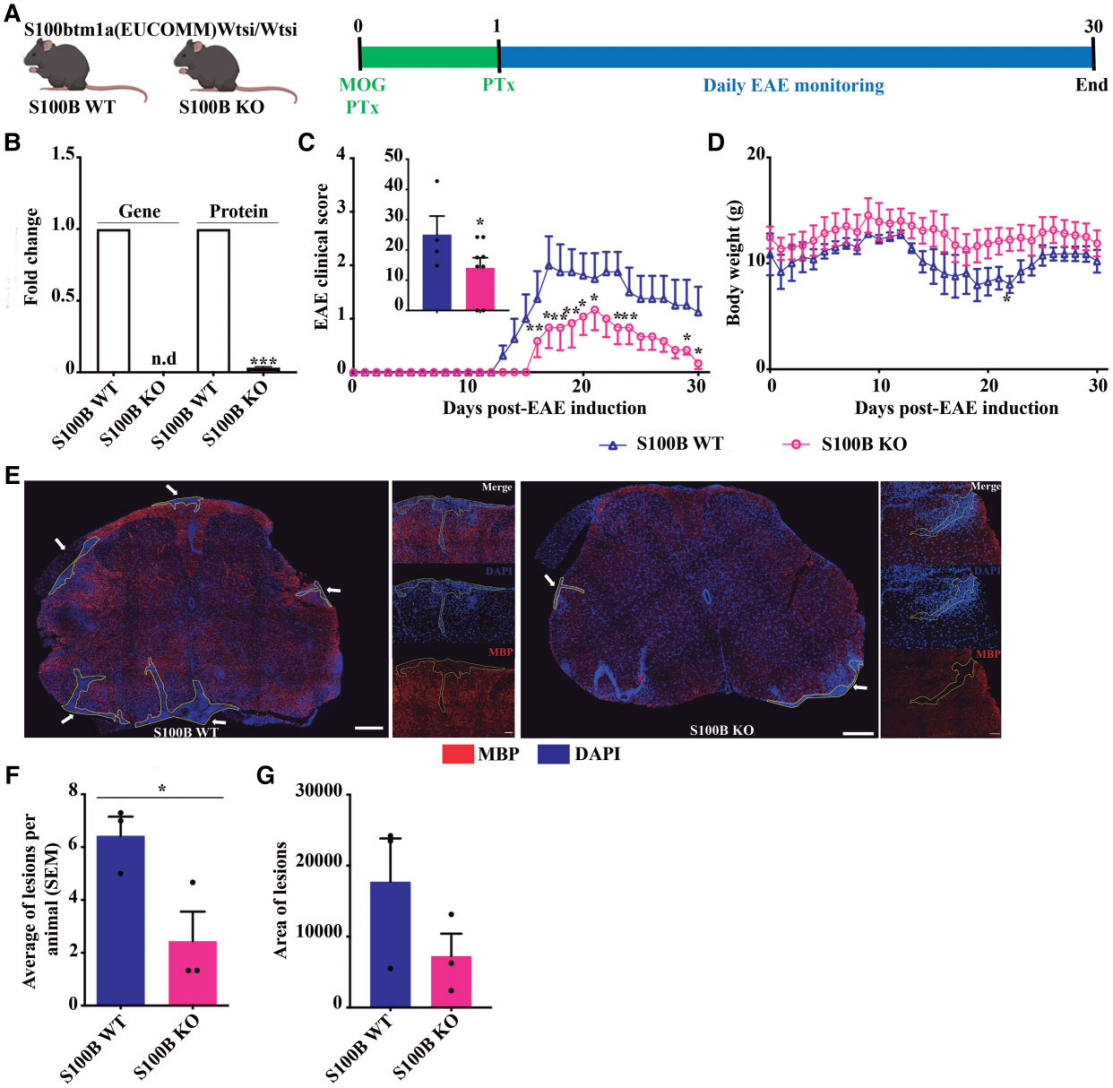
**S100B KO mice are protected from EAE-induced paralysis.** (**A**) Schematic representation of the study outline. Female S100B WT and S100B KO mice were induced with EAE by MOG_35–55_ immunization and monitored until 30 days after EAE induction. (**B**) S100B gene and protein expression levels were determined by qReal-Time PCR and western blot, respectively. (**C**) Clinical score observations and (**D**) body weight were measured for both experimental groups, S100B WT and KO mice, for 30 days. Clinical score was given followed a 5-point scale, establishing a numeric value to the disease severity. The EAE index represents the AUC that was also calculated for each animal. (**E**) Representative images of spinal cord sections showing demyelinating lesions immunostained for mature oligodendrocytes (MBP) and counterstained for cell nuclei (DAPI). Scale bar: 200 µm for slices and 50 µm for insets. Magnification: 20× for slice and 40× for insets. Number of demyelinating lesions and respective area were detected. Unpaired Mann–Whitney *t*-test was used for statistical significance (*P < 0.05, **P < 0.01 and ***P < 0.001 versus S100B WT). (**C**–**D**) *n* = 4 animals for S100B WT, and *n* = 9 animals for S100B KO; (**F**–**G**) *n* = 3 animals for S100B WT and S100B KO.

S100B is increased in the pro-inflammatory context of multiple sclerosis.^4,5,9^ Based on our previous findings^9^ and to explore the importance of S100B in multiple sclerosis development, we used the EAE model, which mimics the inflammation and demyelination of the CNS.^17^ For this, homozygotic (S100B WT) and KO (S100B KO) mice were obtained. After 11 weeks, female mice were EAE-induced by MOG_35–55_ immunization (Fig. 1A). Tissue S100B expression in the S100B KO group was almost non-existent when comparing to the S100B WT group (n.d. for gene expression; *P* < 0.001 for protein expression, Fig. 1B). S100B deletion delayed disease onset, reduced associated paralysis and accelerated animal recovery, mostly in chronic EAE stages (*P* < 0.05; Fig. 1C), with a slight improvement in mice body weight (Fig. 1D). Regarding locomotor ability, in the pole test, no alterations were observed between both conditions before clinical symptoms (8 dpi, time to turn: 5.33 ± 1.53 for S100B WT and 6.14 ± 1.35 for S100B KO; 8 dpi, time to descend: 4.67 ± 0.71 for S100B WT and 5.14 ± 1.86 for S100B KO). Pathologically (Fig. 1E), S100B KO mice showed a significant (*P* < 0.05) reduction in the total number of demyelinating lesions (Fig. 1F), accompanied by a decrease in lesion area (Fig. 1G).

Our data suggest that S100B deletion mitigates some motor symptoms elicited by EAE and prevents myelin degradation and cell infiltration.

### S100B KO mice show reduced CNS glia reactivity and inflammation following demyelination

**Figure 2:**
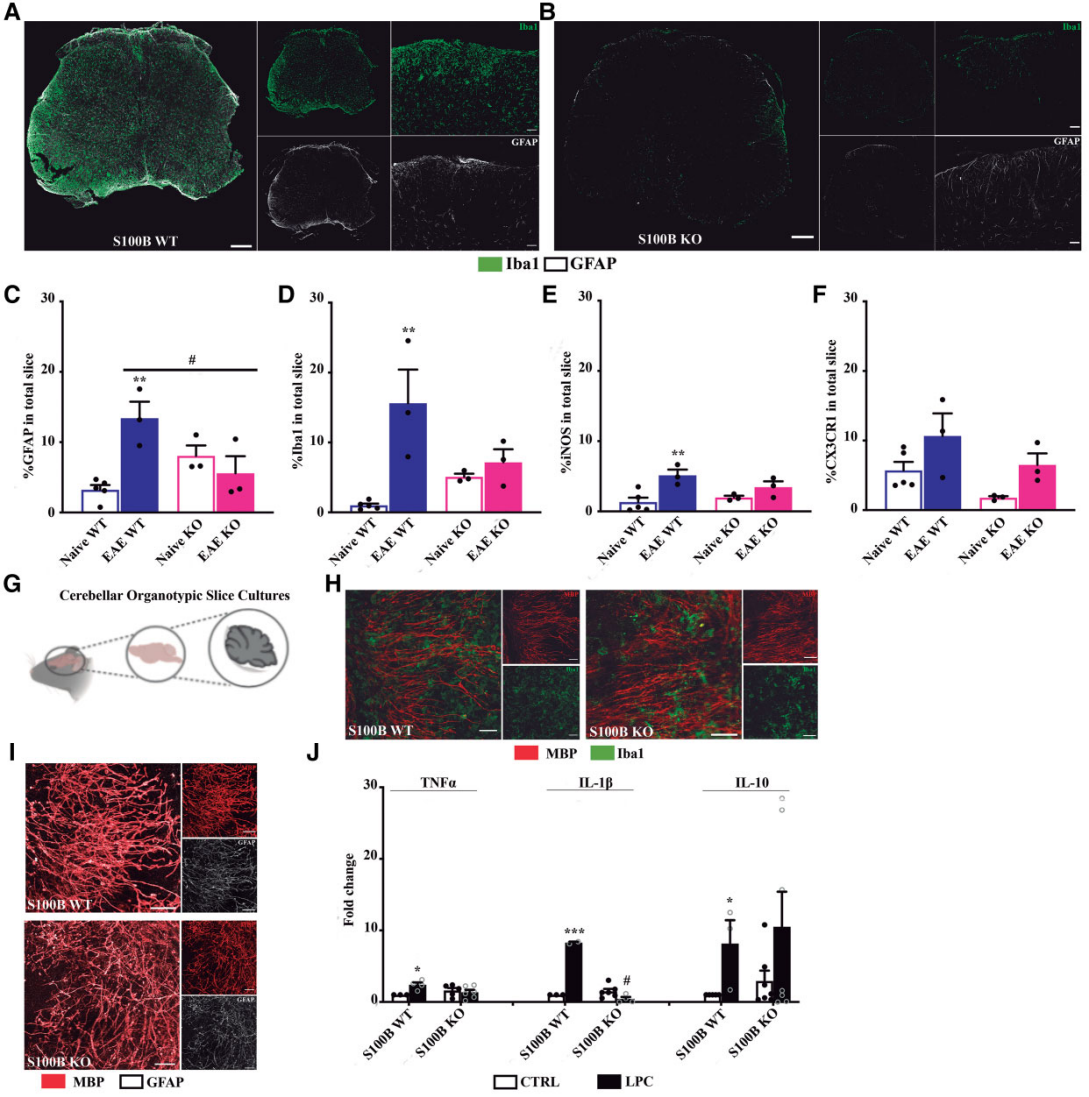
**S100B KO mice present a reduced CNS inflammation in the *in vivo* model of chronic multiple sclerosis and in the *ex vivo* demyelinating model.** Thirty days post-EAE induction, mice were sacrificed, and the spinal cords were collected. Representative images of spinal cord sections immunostained for microglia (Iba1) and for astrocytes (GFAP) for (**A**) EAE WT and for (**B**) EAE KO mice. Scale bar: 200 µm for slices and 50 µm for insets. Magnification: 20× and 40× for insets. Graph bars represent the percentage of area stained for (**C**) astrocytes (GFAP), (**D**) microglia/macrophages (Iba1), (**E**) iNOS and (**F**) CX3CR1 in the total slice of S100B WT and S100B KO groups. (**G**) Organotypic cerebellar slice cultures were exposed to LPC for 18 h and allowed to recover for additional 30 h, being evaluated at 48 h post-LPC incubation. Representative images of cerebellum sections immunostained for (**H**) microglia (Iba1) and for (**I**) astrocytes (GFAP). Scale bar: 50 µm. (**J**) Graph bars represent relative *TNFα*, *IL-1β* and *IL-10* mRNA expression levels determined by qReal-Time PCR. Results were normalized to β-actin. One-way ANOVA with Tukey post-test was used to determined statistical differences (*P < 0.05, **P < 0.01 and ***P < 0.001 versus S100B WT CTRL; ^#^*P* < 0.05 versus respective S100B WT LPC). (**C**–**F**) *n* = 3 animals per group; (**I**): *n* = 3–6 animals for S100B WT and S100B KO.

To demonstrate that S100B deletion protects from EAE by reducing CNS neuroinflammation, immunohistochemistry for astrocytes and microglia/macrophages was analysed in spinal cord sections of EAE-induced S100B WT and KO, and respective naïve animals. As we observe in Fig. 2A and B, reduced expression of both the astrocyte marker GFAP (*P* < 0.05, Fig. 2C) and the microglia/macrophage marker Iba1 (Fig. 2D) was found in EAE KO mice when compared with WT animals. Furthermore, there are no significant differences between naïve KO and EAE KO animals for both markers (Fig. 2C and D). The same does not occur with the WT group. Indeed, we observe a significant increase in both GFAP (*P* < 0.01, Fig. 2C) and Iba1 (*P* < 0.01, Fig. 2D) expression in EAE-induced WT animals when compared to respective naïve.

Then, we also performed immunohistochemistry to unveil microglia phenotypes using iNOS and fractalkine receptor (CX3CR1) markers for pro-inflammatory and homeostatic/phagocytic microglia, respectively. As shown in Fig. 2E, a significant increase is observed in the iNOS expression in the entire slice of EAE WT (*P* < 0.01, Fig. 2E) when compared with naïve WT and a slight but no significant increase in the CX3CR1 expression in the total slice (Fig. 2F). In agreement with the previous data, no changes in iNOS expression were observed in S100B KO animals when compared with the respective naïve group (Fig. 2E), and although CX3CR1 expression was slightly increased in EAE KO animals, no significant changes were obtained versus naïve S100B KO group (Fig. 2F).

Concordantly, demyelination of OCSC by LPC (Fig. 2G) elicited a reduced Iba1 (Fig. 2H) and GFAP (Fig. 2I) immunoreactivity in tissue from S100B KO animals when compared with S100B WT ones. *TNFα* (*P* < 0.05) and *IL-1β* (*P* < 0.001) were significantly increased in S100B WT cultures as well as *IL-10* (*P* < 0.05) after LPC-induced demyelination when compared with respective naïve animals (Fig. 2J). Attractively, in S100B KO cultures, both *TNFα* and *IL-1β* (*P* < 0.05) were downregulated, whereas *IL-10* upregulation was maintained when compared with demyelinated S100B WT cultures (Fig. 2J).

Our data indicate that S100B deletion reverts the glial scenario and the pro-inflammatory profile observed upon a demyelinating insult.

### Neutralization of S100B prevents multiple sclerosis-like pathology in the demyelinating *ex vivo* model

As S100B deletion was able to abrogate CNS neuroinflammation, we hypothesized that S100B blockade could be an effective approach to prevent CNS-associated damage. The therapeutic efficacy of three different S100B blockade strategies (anti-S100B, RAGE antagonist and pentamidine) were compared in the *ex vivo* demyelinating model described previously. Previous and published data from our laboratory^9,12^ were plotted with data from pentamidine studies. S100B levels, oligodendrogenesis, gliosis and inflammation were measured and normalized to naïve group values.

**Figure 3:**
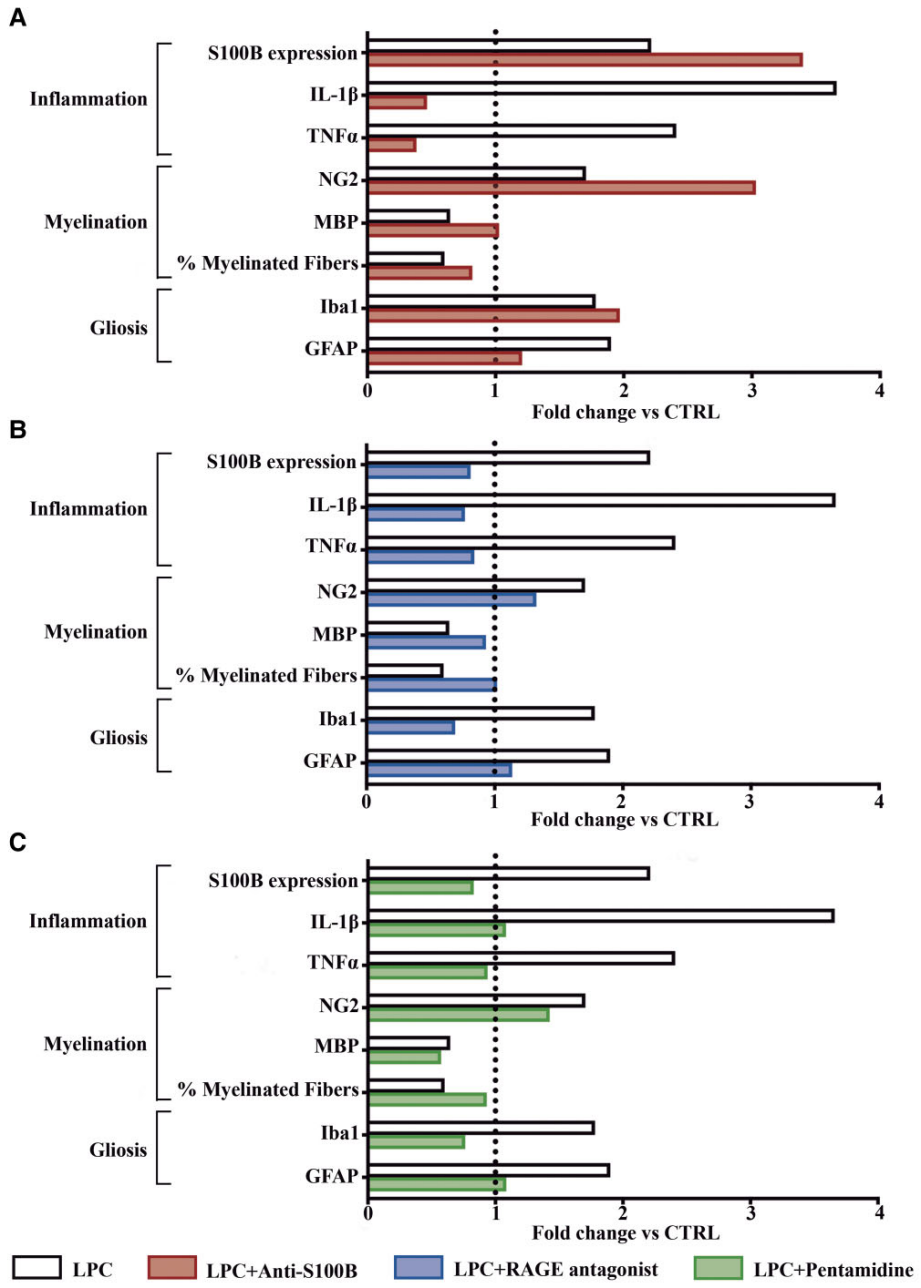
**Neutralization of toxic S100B by different therapeutic approaches using the *ex vivo* model of demyelination.** Each graph represents the therapeutic effects of each treatment. Each parameter was normalized to naïve group values (dashed line). For this, cerebellar organotypic slice cultures were incubated with LPC at 7 day *in vitro* for 18 h to induce demyelination. The different treatments (**A**) antibody anti-S100B, (**B**) RAGE antagonist—FPS-ZM1 and (**C**) pentamidine were incubated at the same time as LPC and maintained for the additional 30 h of recovery. The percentage of myelinated fibers was calculated by the ratio between the area of co-localization of neurofilaments (NF-160) and mature oligodendrocytes (MBP), and the total area occupied by NF-160. Oligodendrogenesis was evaluated by the quantification of precursor cells (NG2) and MBP. Gliosis parameter includes positive cells for astrocytes (GFAP) and for microglia (Iba1). Relative levels of the mRNA expression of S100B and inflammatory cytokines (TNFα and IL-1β) were also determined by qReal-Time PCR.

Along with the increased S100B expression, LPC-induced demyelination led to a clear loss of myelinated fibers and altered oligodendrogenesis, as well as worsening side effects such as inflammation and gliosis when compared with the naïve group. Furthermore, although substantially reducing inflammation, protective effects following antibody treatment (Fig. 3A) may be due to the blockade of excessive S100B present in the extracellular spaces. Particularly, RAGE antagonist (Fig. 3B) and pentamidine (Fig. 3C) treatments were the most beneficial for restoring myelination while reducing both neuroinflammation and S100B expression. These effects could be due to a more broaden effect either at RAGE receptor through inhibition of other ligands binding upon RAGE antagonist treatment, or blockade of S100B effect at both extracellular and intracellular levels following pentamidine use.

Since RAGE is not a sole receptor of S100B and its binding can have striking consequences on different cell types,^18^ we envisioned that pentamidine could be the most promising therapeutics for *in vivo* preclinical studies with a high translational potential for progressive multiple sclerosis clinics.

### S100B inhibition by pentamidine protects from EAE-associated paralysis

**Figure 4:**
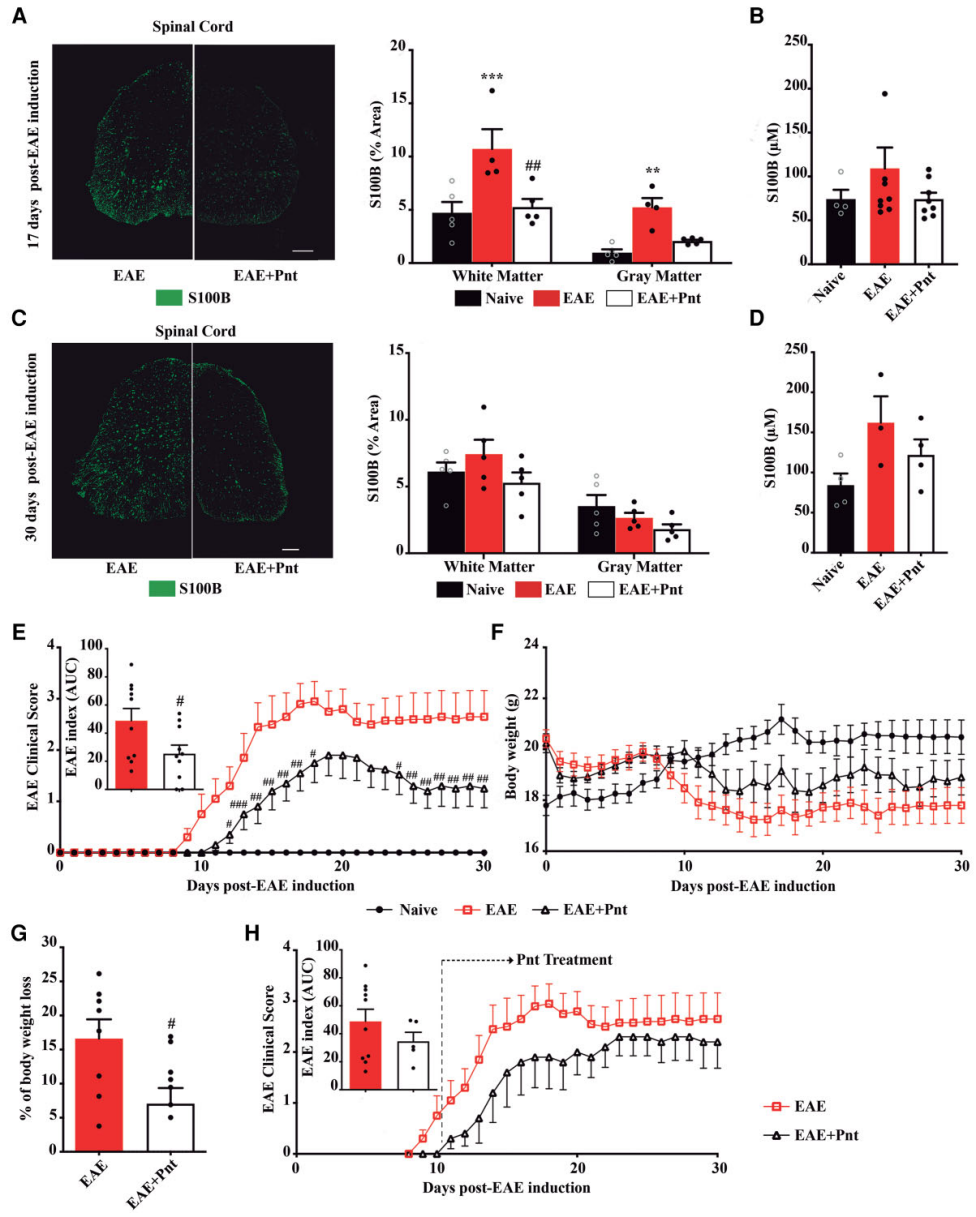
**S100B inhibition by pentamidine prevents its high expression and protects from EAE paralysis.** Female mice were induced with EAE by MOG_35–55_ immunization. Treatment with pentamidine started at day of EAE induction and lasted for 30 days. Representative images of spinal cord sections showing S100B expression and graph bars represent the percentage of area stained for S100B at (**A**) 17 and (**C**) 30 dpi. Scale bar: 200 µm. Magnification: 20×. S100B was determined by ELISA in animals’ serum at (**B**) 17 and (**D**) 30 dpi. (**E**) Clinical observations for prophylactic studies were measured for the experimental groups following a 5-point scale, establishing a numeric value to disease severity. The EAE index is represented by the AUC calculated for each animal. (**F**) Body weight was also measured during the 30 dpi and **(G)** the percentage of body weight was calculated at 30 dpi. (**H**) Clinical observation for therapeutic intervention was measured for the experimental groups following a 5-point scale. The EAE index is represented by the AUC calculated for each animal. Two-way ANOVA with multiple comparisons was used for statistical significance for (**A**) (**P < 0.01, and ***P < 0.001 versus naïve; and ^##^*P* < 0.01 versus EAE). Unpaired Mann–Whitney *t*-test was used for statistical significance for (**E**–**G**) (^#^*P* < 0.05, ^##^*P* < 0.01, and ^###^*P* < 0.001 versus EAE). (**A**–**D**) *n* = 5 animals per group; (**E**–**G**) *n* = 5 animals for naïve, *n* = 10 animals for EAE and EAE + Pnt; H: *n* = 10 animals for EAE and 5 for EAE + Pnt.

First, we characterized S100B expression upon EAE induction. Immunohistochemistry data at 17 and 30 dpi showed a clear transient phenomenon when comparing values between timepoints (Fig. 4). An initial significant increase of S100B expression was observed in white (*P* < 0.001) and grey (*P* < 0.01) matter in non-treated EAE group in comparison to the naïve group. Treatment with pentamidine significantly reverted the effect elicited by EAE induction mainly in the white matter (*P* < 0.01; Fig. 4A). At later stages, 30 dpi, S100B was reduced, with no significant alterations from naïve or pentamidine-treated animals (Fig. 4C). The increased expression of local S100B was then followed by its peripheric release to serum at both timepoints (Fig. 4B–D).

Several *in vivo* studies demonstrated the positive effects of blocking S100B toxic levels using pentamidine in the context of Alzheimer’s disease,^14^ acute colitis^16^ and sepsis.^19^ Here, we clarify the effect of pentamidine as an effective approach to prevent the onset and progression of chronic EAE. For this, pentamidine treatment started on the day of EAE induction and was followed by 30 days. EAE mice typically demonstrated the appearance of motor impairments around 11–13 dpi, reaching the plateau at 16 dpi with a mean score of 3.26 ± 0.37 (Fig. 4E). Pentamidine-treated animals exhibited lower clinical scores, reaching a maximum mean score of 2.20 ± 0.44. Attractively, the appearance of the first clinical signs was significantly delayed in pentamidine-treated animals (13.13 dpi ± 0.69, *P* < 0.05) when compared with non-treated ones (11.10 dpi ± 0.50). In addition, pentamidine significantly reduced the clinical symptoms of chronic EAE at the end of the experiment (*P* < 0.01) with some animals presenting a clinical score equal to zero or one, which corresponds to normal motor function. A significant difference was evident for the area under the curve (AUC), which indicates the combined clinical scores for the entire experimental period (0–30 days), i.e. EAE = 48.88 and EAE + Pnt = 25.41 (*P* < 0.05). Regarding animals’ body weight, the non-treated EAE group suffered a significant loss of body weight that was maintained until the end of the experiment (Fig. 4F). Interestingly, treatment with pentamidine significantly prevented this weight loss (*P* < 0.05) when compared with the non-treated EAE group (Fig. 4G).

To test whether pentamidine strategy can be translationally applied to human multiple sclerosis relapses, we assayed pentamidine treatment on the first day of clinical signs (clinical score equal to 0.5–1) of individual mice. Mirroring the previous preventive studies, pentamidine treatment tends to delay symptoms appearance and mitigate paralysis until disease peak (17 dpi, mean score of 1.9 ± 1.34) when compared with the non-treated EAE group, which was corroborated by a reduction of the EAE index (Fig. 4H).

Overall, our results indicate that pentamidine is effective in reducing paralysis during EAE progression and even in improving animals’ recovery.

### Pentamidine improved EAE-associated motor impairment and reduced the number of demyelinating lesions

**Figure 5:**
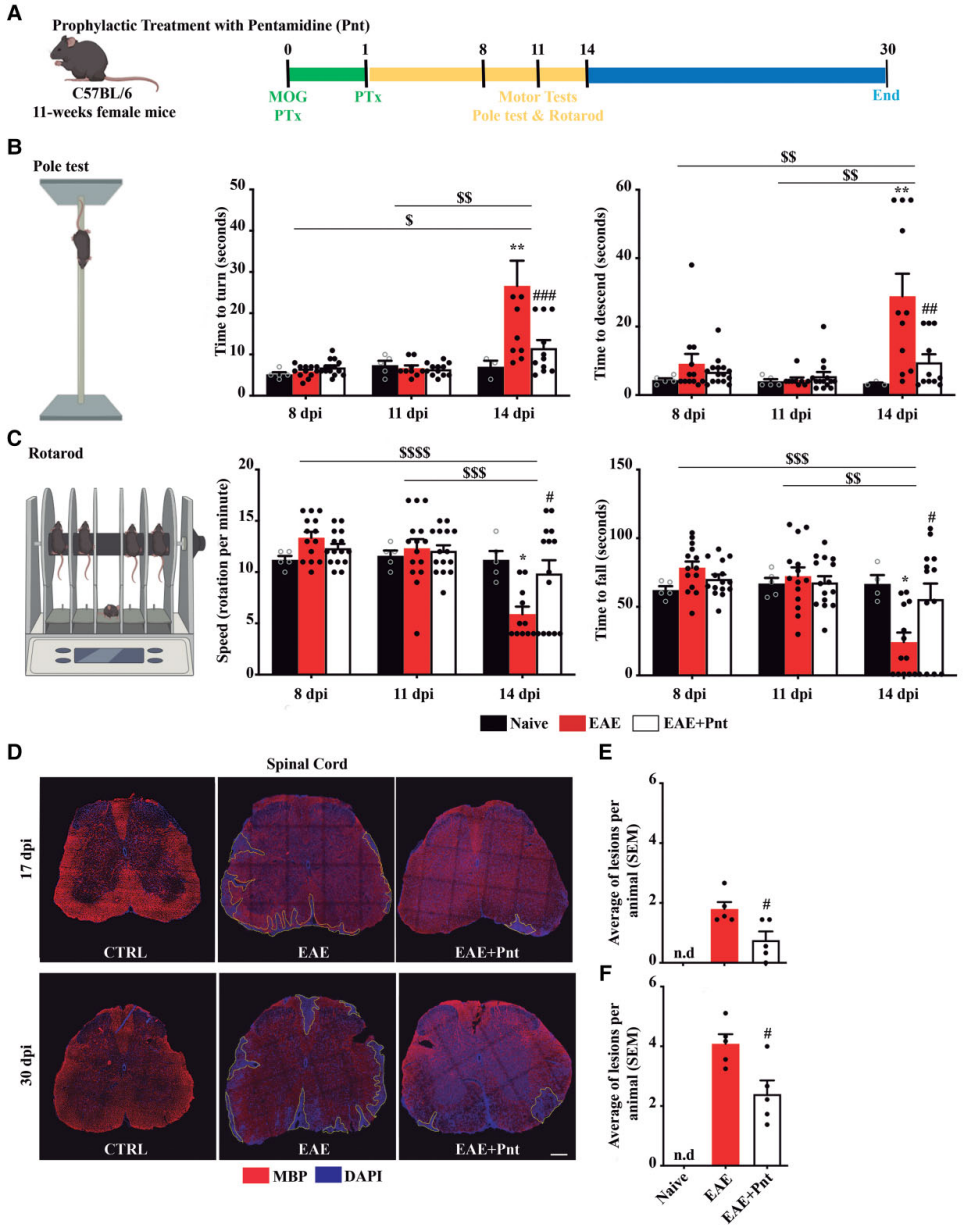
**Pentamidine treatment prevents motor impairment and the formation of new demyelinating lesions during EAE progression.** (**A**) Schematic representation of the study outline. Locomotor tests were performed at 8, 11 and 14 dpi. (**B**) In the pole test, the time to turn and the time to descend the pole were assessed. (**C**) In the rotarod test, the speed of the rod at the time of the fall and the time to fall of the rotating rod were assessed. (**D**) Representative images of spinal cord sections showing delineated demyelinated lesions immunostained for mature oligodendrocytes (MBP) and counterstained for cell nuclei (DAPI). Scale bar: 200 µm. Magnification: 20×. Number of demyelinated lesions were assessed at (**E**) 17 and (**F**) 30 dpi in all experimental groups. One- and two-way ANOVA with multiple comparisons was used for statistical significance (**P < 0.01 versus respective naïve; ^#^*P* < 0.05, ^##^*P* < 0.01, and ^###^*P* < 0.001 versus respective EAE; and ^$^*P* < 0.05, ^$$^*P* < 0.01, ^$$$^*P* < 0.001, ^$$$$^*P* < 0.0001 versus EAE at different timepoints). (**D**–**F**) *n* = 5 animals per group.

To corroborate clinical score data, we evaluated the animal’s locomotor ability through rotarod and pole tests along the course of disease manifestation and prior to the peak of symptoms (i.e. 8, 11 and 14 dpi; Fig. 5A). In the pole test, throughout time, the EAE group started to take longer to turn and walked slower when descending the pole, where the most significant differences occurred at 14 dpi (*P* < 0.01 for time to turn and time to descend), as showed in Fig. 5B. Interestingly, pentamidine treatment significantly prevented motor impairment (*P* < 0.001 versus EAE for time to turn and *P* < 0.01 for time to descend), allowing treated animals to turn and descend faster than the non-treated EAE group. In the rotarod test, upon clinical manifestations (14 dpi), the non-treated EAE animals presented a significant reduction in the time to fall (*P* < 0.05) that was also accompanied by a reduction in the rod speed at fall (*P* < 0.05) when comparing to naïve group (Fig. 5C). Interestingly, once again, pentamidine treatment was able to significantly prevent such effects (*P* < 0.05) improving animal motor abilities.

Along with motor impairment, the EAE model is characterized by the destruction of myelin sheaths resulting in the formation of demyelinating lesions, known as plaques.^20,21^ To assess whether the beneficial effect of pentamidine was associated with the prevention of lesion formation, we analysed the areas of cell infiltrates in spinal cord sections at peak and chronic disease stages (17 and 30 dpi, respectively). As expected, the naïve animals did not display any demyelinated white matter lesions (Fig. 5D). The presence of plaques was detected at both timepoints in non-treated EAE animals, although more pronounced in chronic stages (>4 lesions per animal). Regarding pentamidine treatment, in both 17 and 30 dpi, the animals presented significantly fewer demyelinating lesions (*P* < 0.05) when compared with the non-treated EAE group (Fig. 5E and F).

Altogether, these results demonstrate that pentamidine treatment is able to reduce motor incapacity in parallel with its positive role in spinal cord lesion formation during EAE chronic progression.

### Pentamidine favours oligodendrogenesis at lesion plaque in the chronic EAE phase

Previous results showed that toxic S100B levels affect oligodendrogenesis and demyelination,^22^ which are key pathological hallmarks of EAE and multiple sclerosis.^3^ So, we examined whether the S100B blockade strategy could reduce demyelination and/or improve remyelination processes in the spinal cord sections.

**Figure 6:**
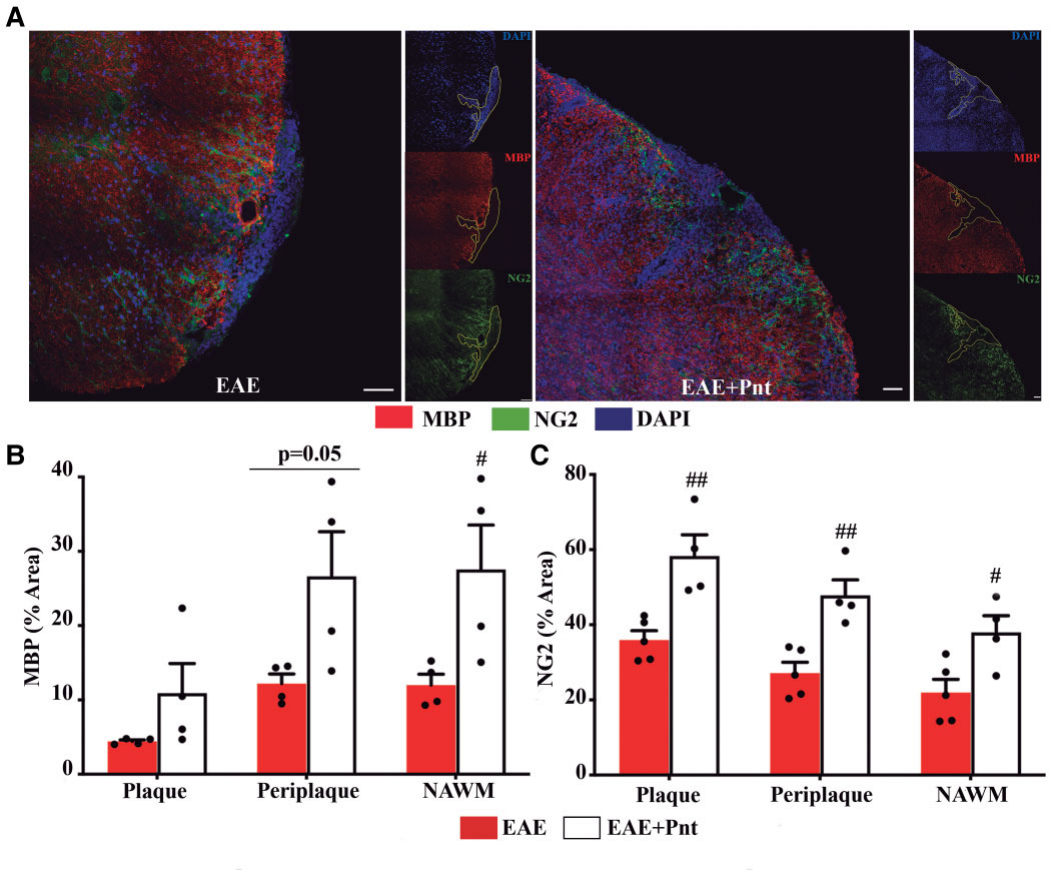
**Pentamidine treatment prevents mature oligodendrocytes loss and induces recruitment of oligodendrocyte precursor cells at chronic EAE stage.** (**A**) Representative images of the three delineated regions: P, PP and NAWM immunostained for mature oligodendrocytes (MBP) and oligodendrocyte progenitor cells (NG2). Scale bar: 50 µm. Magnification: 40×. Graph bars represent the percentage of area stained for (**B**) MBP and (**C**) NG2. The analysis was performed in all experimental groups at 30 days post-EAE induction. Two-way ANOVA with Tukey’s multiple comparisons was used for statistical significance (^#^*P* < 0.05, and ^##^*P* < 0.01 versus EAE) with *n* = 5 animals per group.

An initial loss of mature oligodendrocytes stained for MBP was observed in both white and grey matter of non-treated and pentamidine-treated EAE animals at the peak of disease (Supplementary Fig. 1A); however, this loss became more pronounced at the chronic phase in non-treated EAE animals (*P* < 0.001) while stabilized for the pentamidine group (Supplementary Fig. 2A and B). Thus, at the end-point, the pentamidine group had a significantly increased MBP expression (*P* < 0.05) when compared with the non-treated EAE one (Supplementary Fig. 2A and B). Taking a closer look into the lesion—plaque—and surrounding areas as PP and NAWM, there was a decreased percentage of area stained with MBP that was more pronounced in the plaque and at the disease peak (17 dpi) for both non-treated EAE and pentamidine-treated animals (Supplementary Fig. 1A). However, at a chronic phase, the pentamidine-treated group showed an increased MBP expression mainly at the PP and NAWM (*P* < 0.05; Fig. 6A and B).

The recruitment of oligodendrocyte precursors to the lesion site is essential for remyelination,^23^ so we decided to also evaluate the density of immature oligodendrocytes that are NG2 positive. As expected, at the disease peak (17 dpi) there were no clear differences between EAE and naïve animals, even when lesions and surrounding areas were evaluated in non-treated and pentamidine-treated EAE animals (Supplementary Fig. 1B). Nevertheless, in chronic stages (30 dpi; Supplementary Fig. 2C), there was a massive increase of NG2 density in both white and grey matter of non-treated and pentamidine-treated animals (*P* < 0.01) that was even significantly higher at plaque (*P* < 0.01), PP (*P* < 0.01) and NAWM (*P* < 0.05) of the pentamidine-treated group (Fig. 6A and C).

These results demonstrate that pentamidine prevents oligodendrogenesis impairment inducing the recruitment of progenitor’s cells mainly to lesion plaque area.

### Pentamidine partially decreases EAE-associated astrogliosis but enhances microglia/macrophages recruitment

To study the impact of elevated S100B levels in gliosis, immunohistochemistry for astrocytes and microglia/macrophages was analysed in spinal cord sections.

**Figure 7:**
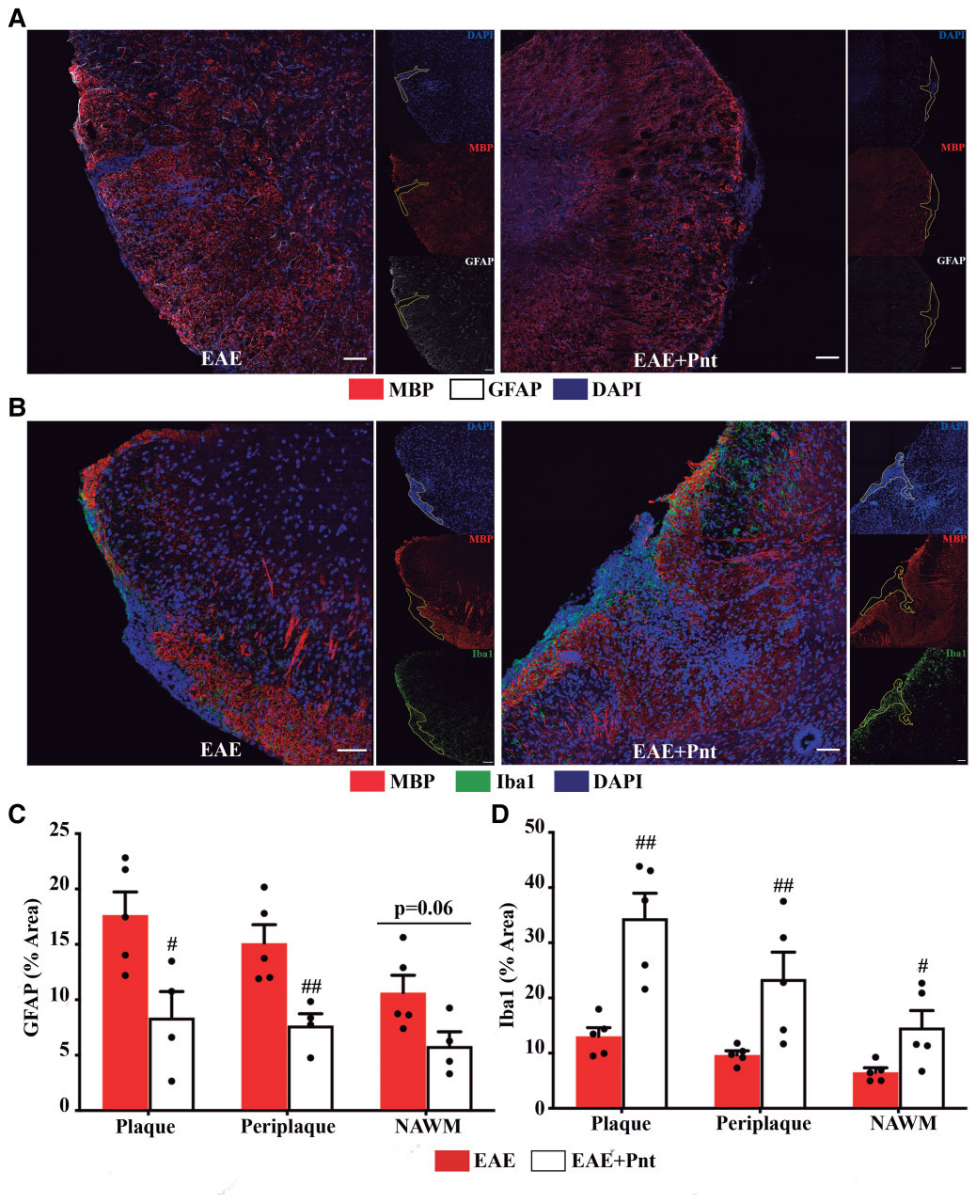
**Pentamidine treatment partially reduce astroglial reactivity and enhances microglia/macrophage recruitment at chronic EAE phase.** (**A**) Representative images of the three delineated regions: P, PP and NAWM immunostained for astrocytes (GFAP) and microglia/macrophages (Iba1). Scale bar: 50 µm. Magnification: 40×. Graph bars represent the percentage of area stained for (**B**) GFAP and (**C**) Iba1. The analysis was performed in all experimental groups at 30 days post-EAE induction. Two-way ANOVA with Tukey’s multiple comparisons was used for statistical significance (^#^*P* < 0.05 and ^##^*P* < 0.01 versus EAE) with *n* = 5 animals per group.

At EAE peak, 17 dpi, there was a significant increase of GFAP density in the non-treated EAE group in both white (*P* < 0.05) and grey matter (*P* < 0.01) that was almost completely abrogated by pentamidine treatment (*P* < 0.05; Supplementary Fig. 1C). This inhibitory effect was mainly observed at PP and NAWM regions (*P* < 0.05). The astrogliosis was slightly exacerbated in the chronic disease phase, 30 dpi, namely for the pentamidine-treated animals (*P* < 0.01 versus naïve; Supplementary Fig. 3A and B), although at the lesion site it was still observed a significant suppressive effect of pentamidine at plaque (*P* < 0.05) and PP (*P* < 0.01) regions (Fig. 7A and C).

Alongside, EAE induction elicited an increase in microglia/macrophage density at disease peak, 17 dpi, that was significant for the grey matter of non-treated EAE animals (*P* < 0.05) (Supplementary Fig. 1D), and higher at plaque region. This microgliosis was reduced at the chronic phase, 30 dpi, for non-treated EAE animals, although still significant for white matter when compared with naïve animals (*P* < 0.05; Supplementary Fig. 3C). Intriguingly, at this phase pentamidine-treated animals showed a highly enhanced microglia/macrophage density mainly in white (*P* < 0.001 versus naïve) and in grey matter (Supplementary Fig. 3C) that was significantly augmented when compared with non-treated animals at plaque (*P* < 0.01), PP (*P* < 0.01) and NAWM (*P* < 0.05; Fig. 7B and D).

To further decipher which type of microglia/macrophages were induced upon pentamidine treatment, we evaluated the expression of pro-inflammatory iNOS and the resolution of damage CX3CR1-positive cells. We observed a marked increase of iNOS expression in non-treated EAE animals at disease peak, 17 dpi (Supplementary Fig. 4A), that was maintained in the chronic phase, 30 dpi (Supplementary Fig. 4B), when compared with naïve animals (*P* < 0.001 for white matter; *P* < 0.01 for grey matter). Curiously, pentamidine treatment was able to prevent EAE-induced iNOS expression at both timepoints, with a more marked action at 30 dpi in both white (*P* < 0.01) and grey (*P* < 0.05) matter (Supplementary Fig. 4B), and at plaque (*P* < 0.01), PP (*P* < 0.05) and NAWM (*P* < 0.05) regions (Supplementary Fig. 4C and D). In addition, non-treated EAE group showed increased serum levels of TNFα and IL-1β when compared with the naïve group (*P* < 0.05; Supplementary Fig. 4E and F) that was significantly reduced in the pentamidine-treated group (*P* < 0.05) corroborating the previous results.

Regarding CX3CR1 staining, the differences between groups were mainly observed at chronic stages. A slight increase of CX3CR1 expression was observed in non-treated EAE animals at disease peak (Supplementary Fig. 5A) that was maintained throughout time (Supplementary Fig. 5B). Interestingly, this increase was significantly enhanced by pentamidine treatment at 30 dpi in both white (*P* < 0.001) and grey matter (*P* < 0.001). Although with no alterations at 17 dpi (Supplementary Fig. 5C), we observed the same significant increase in pentamidine-treated animals at lesion plaque (P < 0.01) and surrounding areas (P < 0.001) when comparing to non-treated EAE group (Supplementary Fig. 5D).

These data are indicative of pentamidine efficacy in ameliorating CNS neuroinflammation by reducing astrocytic reactivity and inducing a less inflammatory and more regenerative/phagocytic microglia in demyelinating lesions.

### Pentamidine enhances CNS infiltration of regulatory T cells following EAE induction

Increased reactivity of immune cells (e.g.Th1/Th17), defective function of Treg cells and the presence of inflammatory cytokines are key players in multiple sclerosis and EAE pathology.^24,25^ Next, we examined whether pentamidine treatment could modulate the infiltration of immune cells at the spinal cord.

**Figure 8:**
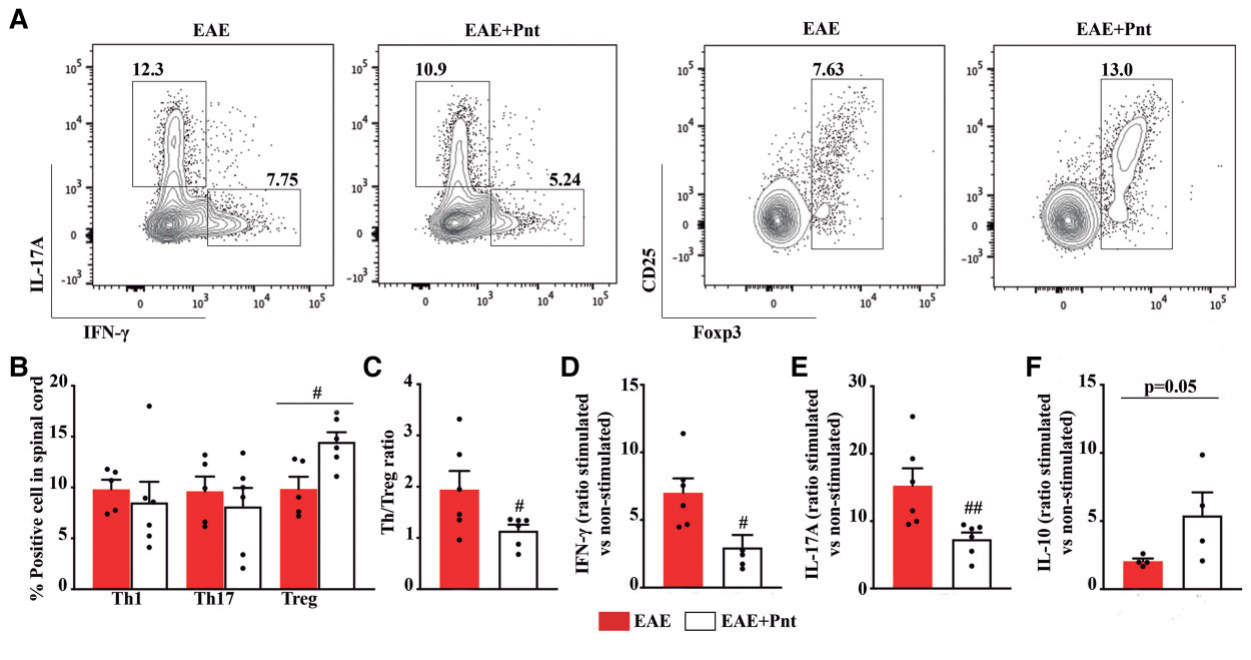
**Pentamidine treatment enhances a spinal cord T cell regulatory phenotype.** Seventeen days post-EAE induction, spinal cord mononuclear cells were stimulated *ex vivo* with PMA/ionomycin in the presence of protein secretion inhibitors and the expression of IFN-γ, IL-17A and Foxp3 was evaluated by intracellular flow cytometry. (**A**) Representative contour plots, (**B**) percentage of positive cells within parent population and (**C**) Th/Treg ratio are shown. Sorting strategy is described in Supplementary Fig. 6. At the same timepoint, cells from cervical lymph nodes were stimulated *ex vivo* with MOG_35–55_ and (**D**) IFN-γ, (**E**) IL-17A and (**F**) IL-10 were quantified in the culture supernatants after 72 h by ELISA. Graph bars represent the ratio between stimulated and non-stimulated conditions. Unpaired Mann–Whitney *t*-test was used for statistical significance (^#^*P* < 0.05, and ^##^*P* < 0.01 versus EAE) with *n* = 5–7 animals per group.

At EAE peak, pentamidine slightly reduced the number of Th cell populations that express IFN-γ, the Th1 and IL-17A, the Th17 (Fig. 8A and B), although with no alteration on infiltrating CD3^+^ population, identified in accordance with the gating strategy (Supplementary Fig. 6A and B). However, CD4 ^+^ Foxp3^+^ Treg density at the spinal cords of pentamidine-treated animals was significantly higher (*P* < 0.05, Fig. 8A and B) resulting in a significant decrease in Th/Treg ratio (*P* < 0.05, Fig. 8C) when compared with the non-treated EAE group. To further corroborate these data, we also evaluated cytokines released *in vitro* by the restimulation of immune cells isolated from cervical lymph nodes with MOG_35–55_ peptide. The ratio between stimulated and non-stimulated immune cells from the treated EAE group showed a significant decrease in IFN-γ (*P* < 0.05, Fig. 8D) and IL-17A (*P* < 0.01, Fig. 8E), whereas a slight increase in IL-10 (*P* = 0.05, Fig. 8F) when compared with the non-treated EAE.

Our data indicated that pentamidine affects immune tolerance promoting a more regulatory and less inflammatory response.

## Discussion

Here, we describe for the first time the role of S100B in chronic EAE development and how its therapeutic blockade with pentamidine has beneficial outcomes in multiple sclerosis-like CNS- and immune pathology in chronic EAE mice. Indeed, we show that S100B is a crucial contributor to EAE and spinal cord pathology. Furthermore, we found that S100B blockade by pentamidine in the chronic EAE model acts by preventing lesion formation, neuroinflammation and modulating immune infiltration, thus reducing disease progression and fastening recovery. Our preclinical data reinforce the involvement of S100B in pathology and the possibility to repurpose pentamidine as a novel potential therapy for multiple sclerosis.

DAMPs, or alarmins, comprise a set of endogenous molecules (e.g. S100B) that are released to the extracellular space upon tissue injury.^26^ Cells of the innate immune system recognize and respond to these warming signals through specific receptors (e.g. RAGE) which, depending on the cellular context, can stimulate cell differentiation or death, or secretion of inflammatory mediators.^7,26^ Particularly, toxic S100B levels have been directly related to disease progression as in acute brain injury,^27^ congenital/perinatal disorders,^28^ psychiatric disorders^29^ and neurodegenerative diseases including multiple sclerosis.^30^ Consequences of excessive S100B in the CNS include the following: (i) neuronal death by reactive oxygen species activation;^31^ (ii) microglial and astrocyte activation^32,33^ and (iii) impaired oligodendrogenesis.^22^ Previously, we have shown that S100B is upregulated in both CSF and serum in multiple sclerosis diagnosis, as well as in the active multiple sclerosis lesions from post-mortem tissues.^9^ In addition, S100B is highly overexpressed and released in demyelinating conditions, validating the disease models used in the present study.^9^ We found that S100B depletion mitigates motor symptoms associated with clinical EAE and decreased cell infiltration, thus preventing the formation demyelinating lesions. Furthermore, our results show that S100B depletion from the beginning of EAE prevented astrocyte and microglia/macrophages activation together with an increased cell population expressing CX3CR1 resulting in decreased TNFα and IL-1β gene expression. This is known to be mediated by the RAGE receptor after overexpression of S100B, which leads to the activation of the nuclear factor (NF)-κB pro-inflammatory cascade.^34,35^ In addition, S100B ablation was already described to reduce gliosis in other pathologies,^36^ confirming the beneficial outcomes and emphasizing the pejorative effect of S100B on CNS inflammation.

Given the significance of S100B as a therapeutic target, some S100B blockade strategies as the anti-S100B, RAGE antagonist and S100B inhibitors have been studied using the *ex vivo* demyelinating models. Particularly, recent studies demonstrated that inhibiting S100B synthesis through arundic acid was beneficial in lowering disease severity and in reducing demyelination and astrocytosis, reinforcing the role of S100B in chronic EAE.^13^ All strategies have shown promising results, but pentamidine, an approved drug by the Food and Drug Administration and the European Medicines Agency,^37,38^ was very appealing given its potential repurposing use. Attractively, pentamidine has also been shown to reduce inflammation in an Alzheimer’s disease mouse model^14^ and, more recently, to partially improve paralysis in relapsing–remmiting EAE.^15^ We demonstrated that pentamidine not only delayed disease onset in chronic EAE but also reduced disease incidence, in a more marked fashion than previously reported for relapsing–remmiting EAE.^15^ The affinity and high specificity of pentamidine to inhibit S100B activity by blocking the interaction of Ca^2+^/p53 site^39^ may explain the better disease outcomes, although additional mechanisms should be explored. Moreover, the potential toxicity of pentamidine in everyday use for long periods^40^ must be taken into consideration. In addition, pentamidine was also effective when given at the time of EAE onset highlighting its potential usefulness as a new pharmacological strategy for relapses, showing a broad therapeutic action and reduced adverse effects than the standard of care methylprednisolone.^41^ Importantly, although the most prevalent form of multiple sclerosis is the relapsing–remitting disease, patients frequently shift their form to a progressive one where there are no effective therapeutic options. So, our data show promising results in the use of pentamidine for chronic/progressive forms of multiple sclerosis being the first step towards clinical trials and further human application of pentamidine.

In the CNS, S100B is known to have a dual role.^42^ Under EAE induction, S100B is upregulated, thus activating astrocytes and microglia, and creating a pro-inflammatory environment that may favour demyelination and paralysis. We showed that pentamidine treatment reduced the formation of white matter demyelinating lesions and induced oligodendrogenesis at chronic stages. These results confirm the consequences of excessive S100B levels in impairing oligodendrogenesis^22^ and in delaying *de novo* myelination. Nonetheless, it is important to preserve basal S100B for proper differentiation of precursor cells into myelinating mature oligodendrocytes. Indeed, it was demonstrated that the total absence of S100B reduced/delayed oligodendrocyte maturation in primary mixed glial cultures, whereas elicited a lower maturation rate of newly generated oligodendrocyte precursor cells in the *in vivo* cuprizone model.^43^

S100B is actively released by astrocytes under demyelinating circumstances behaving as a DAMP^9^ and their contribution to disease pathology has already been studied during multiple sclerosis^44,45^ and in early EAE, as observed in the present paper and by others.^46^ We demonstrated that astrocytic reactivity was prevented in the demyelinated lesions and surrounding areas confirming pentamidine’s potential beneficial effects on preventing glial scar formation. Prevention of astrocyte migration and proliferation was previously reported following S100B silencing through a reduced activity of the Src kinase/phosphatidylinositol 3-kinase (PI3K) pathway.^47^ Furthermore, reduced astrogliosis by pentamidine was already described in other pathologies and revealed to attenuate hippocampal gliosis via RAGE-dependent manner.^14,19^

In parallel to increased S100B, we have shown an increase of microglia proliferation and activation during demyelination^9^ via RAGE engagement.^9,34^ Concordantly, we observed an increase of microglia/macrophage population during the EAE course, whereas pentamidine enhanced their recruitment to the lesions or near the proximity of plaques, which is considered a multiple sclerosis-related pathological hallmark.^48^ Alongside pentamidine ameliorated the pro-inflammatory scenario by reducing iNOS expression, which was already described in a mouse model of Alzheimer’s disease,^14^ and increasing CX3CR1-producing cells. Importantly, several studies have been demonstrating the importance of CX3CR1 in neurodegeneration. Indeed, knocking out CX3CR1 significantly impeded myelin clearance, leading to the persistence of myelin debris further inhibiting proper remyelination in the *in vivo* demyelinating model.^49^ These data suggest that the presence of microglia/macrophages in the site of injury is related to their crucial functions as phagocytic and regenerative cells and not with their pathogenic functions. Indeed, we modulated microglia towards a more phagocytic state that is able to clear the over-accumulated myelin-toxic debris, which is essential for proper remyelination.^50^

In EAE, the blood–brain barrier’s leakage allows the infiltration of activated lymphocytes and the recruitment of inflammatory cells to the CNS, leading to increased disease severity.^3^ We found that pentamidine promoted Treg cell spinal cord infiltration with an IL-10-producing phenotype, possibly favouring the recovery of treated animals. Moreover, the responsiveness of immune cells from draining lymph nodes to MOG_35–55_ restimulation was altered in pentamidine-treated animals reinforcing that pentamidine potentiates immune regulatory mechanisms at the peripheral level and therefore reduces the EAE-associated inflammatory response. Attractively, we first described a new immunomodulatory property of pentamidine; however, further studies are needed to clearly understand the underlying mechanisms. Our results are in line with a previous study in myasthenia gravis where S100B-mediated RAGE activation exacerbated the disease by enhancing T cell pro-inflammatory responses and aggravating T helper subset imbalance.^51^ Therefore, modulation of S100B expression at both the CNS and peripheral level may be a beneficial therapeutic strategy to reduce multiple sclerosis-associated pathology.

Although a variety of drugs are available in the clinics, it is demanding to identify new therapeutic targets and continue the pursuit for safer and effective drugs for multiple sclerosis treatment, namely for the short periods of relapses and progressive forms. Together, pentamidine may be used as a repurposing and innovative therapeutic strategy and showed to be effective for chronic phenotypes or for clinical relapses where inflammation is exacerbated and the blood–brain barrier is highly permeable allowing the drug’s entrance. Our study unveiled and explored the importance of the S100B molecular targeting through pentamidine as a positive strategy to prevent multiple sclerosis-driven neuroinflammation, promote remyelination and therefore fasten recovery.

## Supplementary Material

fcac076_Supplementary_Data

## Abbreviations

AUC =: area under the curve
cDNA =: complementary DNA
CFA =: complete Freund’s adjuvant
CSF =: cerebrospinal fluid
CX3CR1 =: CX3chemokine receptor 1
DAMP =: damage-associated molecular pattern
DIV =: days *in vitro*
dpi =: days post-EAE induction
EAE =: experimental autoimmune encephalomyelitis
ELISA =: enzyme-linked immunosorbent assay
FITC =: fluorescein isothiocyanate
GFAP =: glial fibrillary acidic protein
HEPES =: 4-(2-hydroxyethyl)-1-piperazineethanesulfonic acid
Iba1 =: ionized calcium-binding adapter molecule 1
IFN =: interferon
IL =: interleukin
iNOS =: inducible nitric oxide synthase
LPC =: lysophosphatidylcholine
MBP =: myelin oligodendrocyte glycoprotein
MOG =: myelin oligodendrocyte glycoprotein
NAWM =: normal-appearing white matter
NG2 =: neuron-glial antigen 2
OCSC =: organotypic cerebellar slice cultures
P =: plaque
PCR =: polymerase chain reaction
Pnt =: pentamidine
PP =: periplaque
Ptx =: pertussis toxin
RAGE =: receptor for advanced glycation endproducts
Th =: T helper
TNF =: tumour necrosis factor
Treg =: regulatory T cells
