# Supplementary material for: S100B inhibition protects from chronic experimental autoimmune encephalomyelitis
Source: Brain Commun. 2022 Mar 25;4(3):fcac076. doi: 10.1093/braincomms/fcac076 (PMC9128388; doi:10.1093/braincomms/fcac076)
Supplement: fcac076_Supplementary_Data [file fcac076_Supplementary_Data.docx]

**Supplementary Material**

**
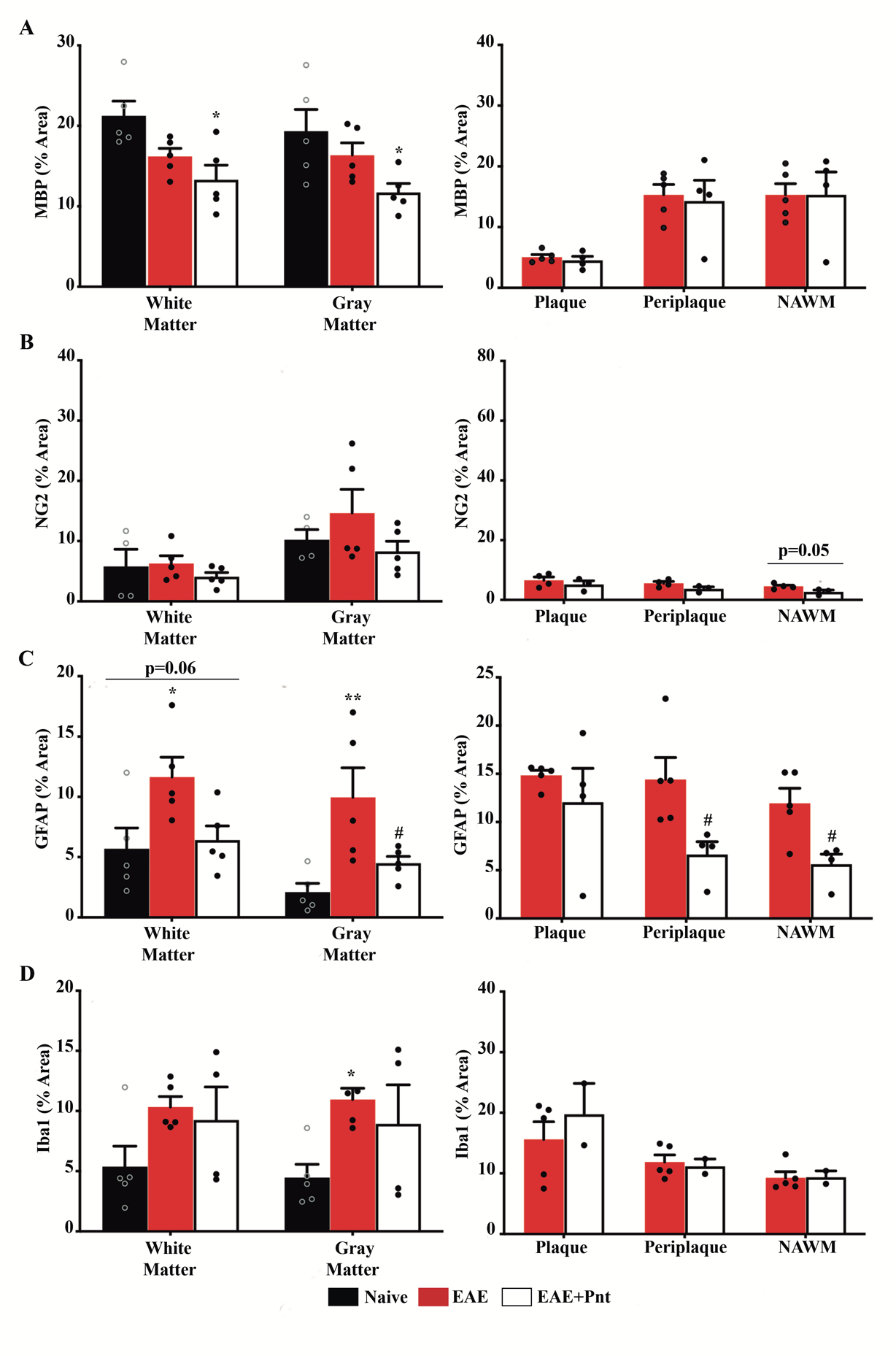
**

**Supplementary Figure 1 -** **Pentamidine treatment differently affect myelination and glia reactivity at EAE peak.** Graph bars represent the percentage of area stained for **(A)** mature oligodendrocytes (myelin binding protein, MBP), **(B)** oligodendrocyte precursor cells (neuron-glial antigen 2, NG2), **(C)** astrocytes (glial fibrillary acidic protein, GFAP) and **(D)** microglia (ionized calcium-binding adaptor molecule 1, Iba1). The analysis was performed in spinal cord sections of all experimental groups at 17 days post-EAE induction, either in white and gray matter or in Plaque (P), Periplaque (PP) and Normal Appearing White Matter (NAWM). Two-way ANOVA with Tukeys multiple comparisons was used for statistical significance (**P<*0.05, and ***P<*0.01 vs naïve; #*P<*0.05 vs EAE) with n = 5 animals per group.

**
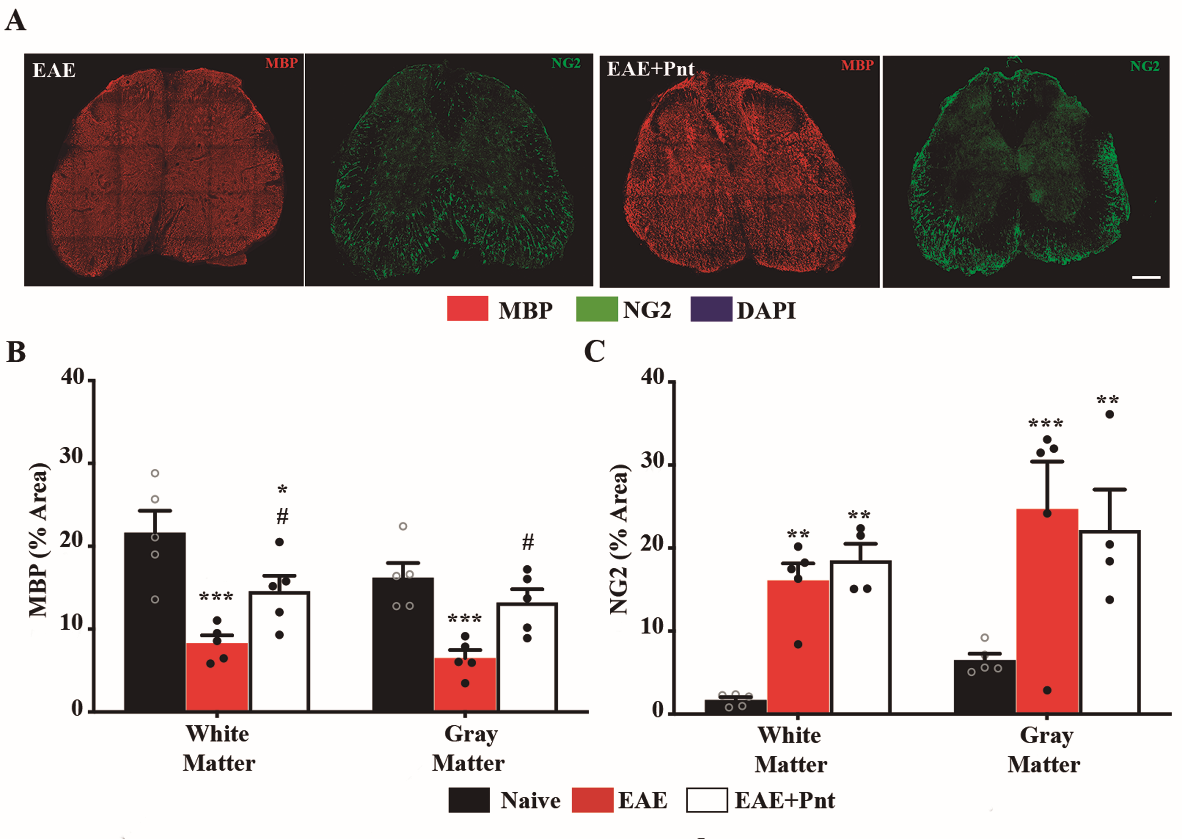
**

**Supplementary Figure 2** - **Pentamidine treatment prevents mature oligodendrocytes loss and induces recruitment of oligodendrocyte precursor cells at chronic-EAE stage.** (**A)** Representative images of spinal cord sections immunostained for mature oligodendrocytes (MBP, red) and for oligodendrocyte progenitor cells (NG2, green). Scale bar: 200 µm. Magnification: x20. Graph bars representing the percentage of area stained for **(B)** MBP and **(C)** NG2. The analysis was performed in all experimental groups at 30 days post-EAE induction. Two-way ANOVA with Tukeys multiple comparisons was used for statistical significance (**P<*0.05, ***P<*0.01, and ****P<*0.001 vs naïve; #*P<*0.05 vs EAE) with n = 5 animals per group.


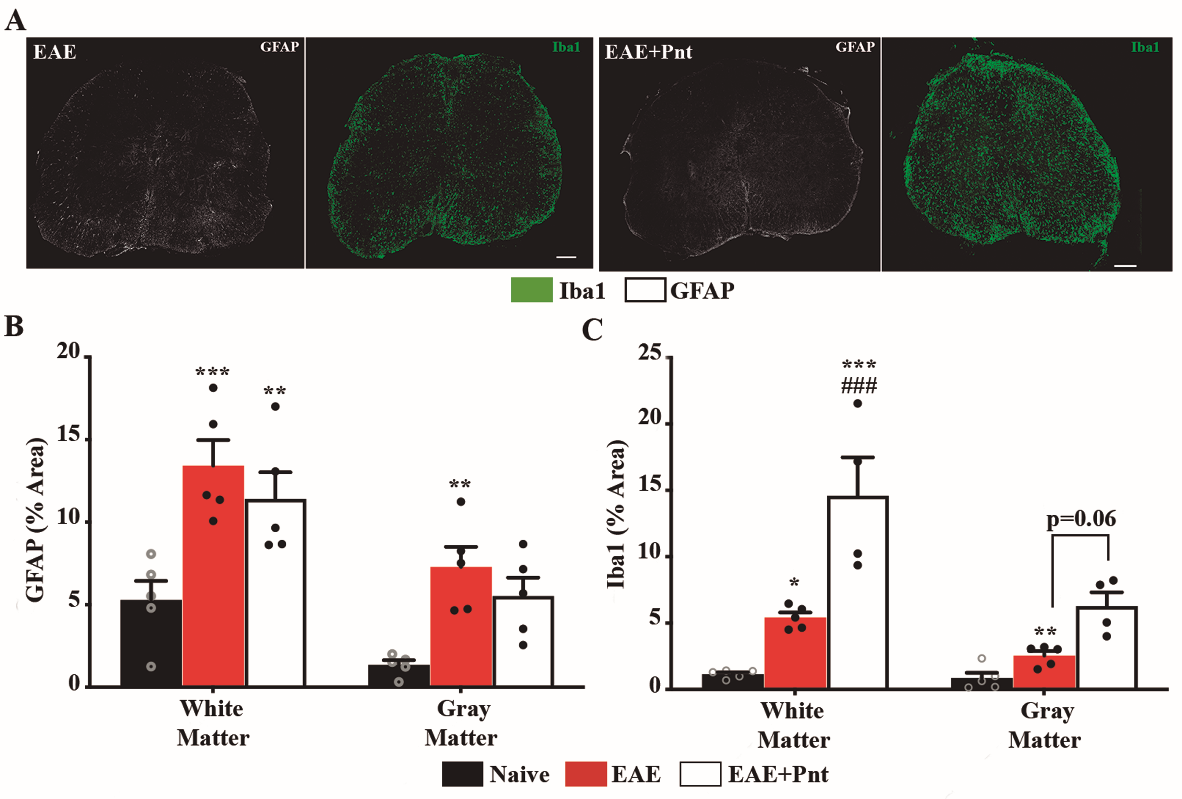


**Supplementary Figure 3 - Pentamidine treatment partially reduce astroglial reactivity and enhances microglia/macrophage recruitment at chronic-EAE phase.** (**A)** Representative images of spinal cord sections immunostained for astrocytes (GFAP, white) and for microglia/macrophages (Iba1, green). Scale bar: 200 µm. Magnification: x20. Graph bars representing the percentage of area stained for **(B)** GFAP and **(C)** Iba1. The analysis was performed in all experimental groups at 30 days post-EAE induction. Two-way ANOVA with Tukeys multiple comparisons was used for statistical significance (**P<*0.05, ***P<*0.01, and ****P<*0.001 vs naïve; ###*P<*0.001 vs EAE) with n = 5 animals per group.

**
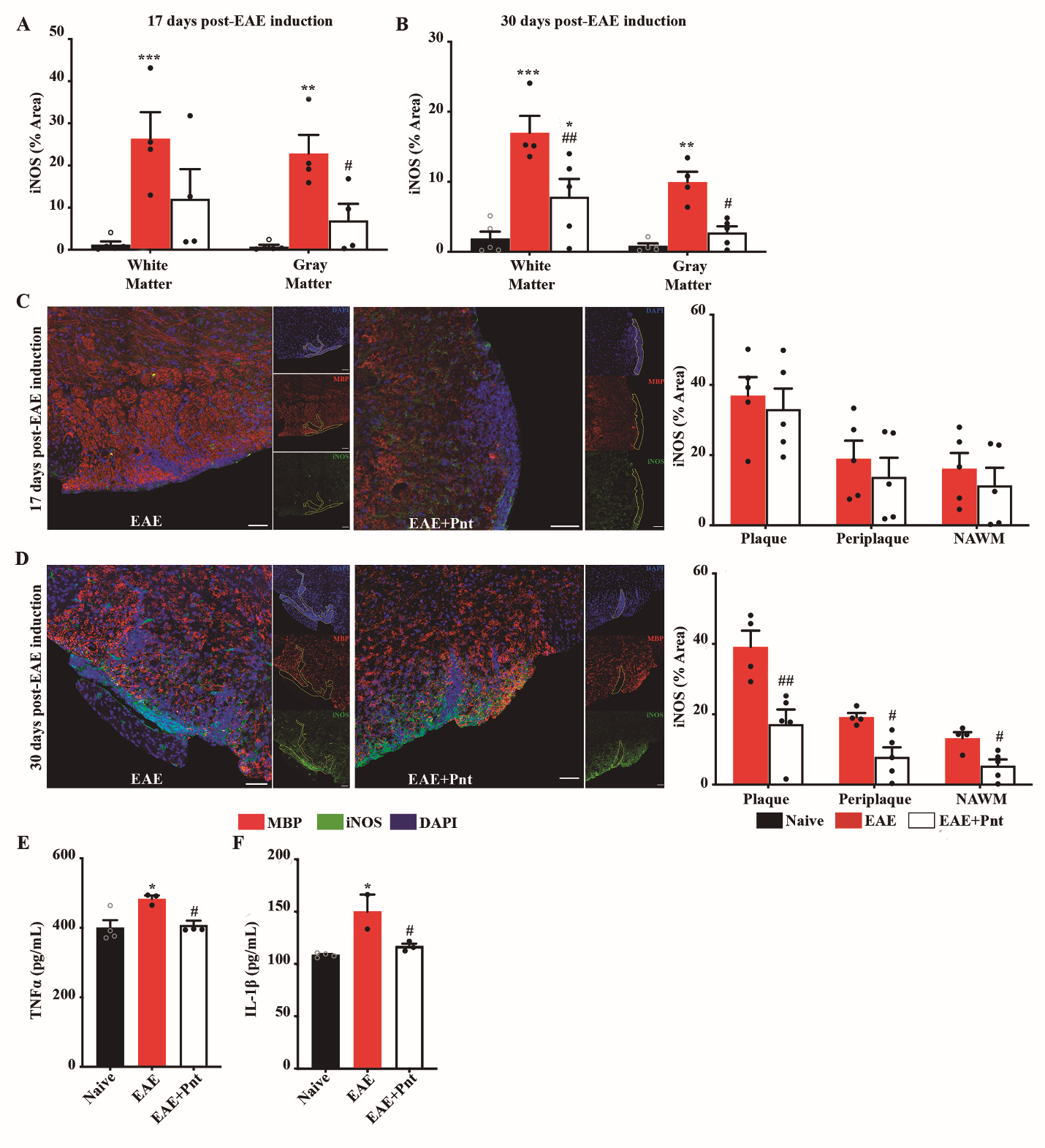
**

**Supplementary Figure 4 - Pentamidine treatment prevents iNOS immunoreactivity and serum pro-inflammatory cytokine enhanced levels associated with EAE.** Graph bars representing the percentage of area stained for inducible nitric oxide synthase (iNOS) at **(A)** 17 and **(B)** 30 days post-EAE induction (dpi). Representative images immunostained for iNOS (green) of the three delineated regions: Plaque (P), Periplaque (PP) and Normal Appearing White Matter (NAWM) and respective quantification at **(C)** 17 and **(D)** 30 dpi. Scale bar: 50 µm. Magnification: x40. **(E)** TNFα and **(F)** IL-1β serum levels were measured by ELISA at 30 dpi. One- and two-way ANOVA with Tukeys multiple comparisons was used for statistical significance (*P<0.05, **P<0.01, and ***P<0.001 vs naïve; #P<0.05, and ##P<0.01 vs EAE) with n = 5 animals per group.


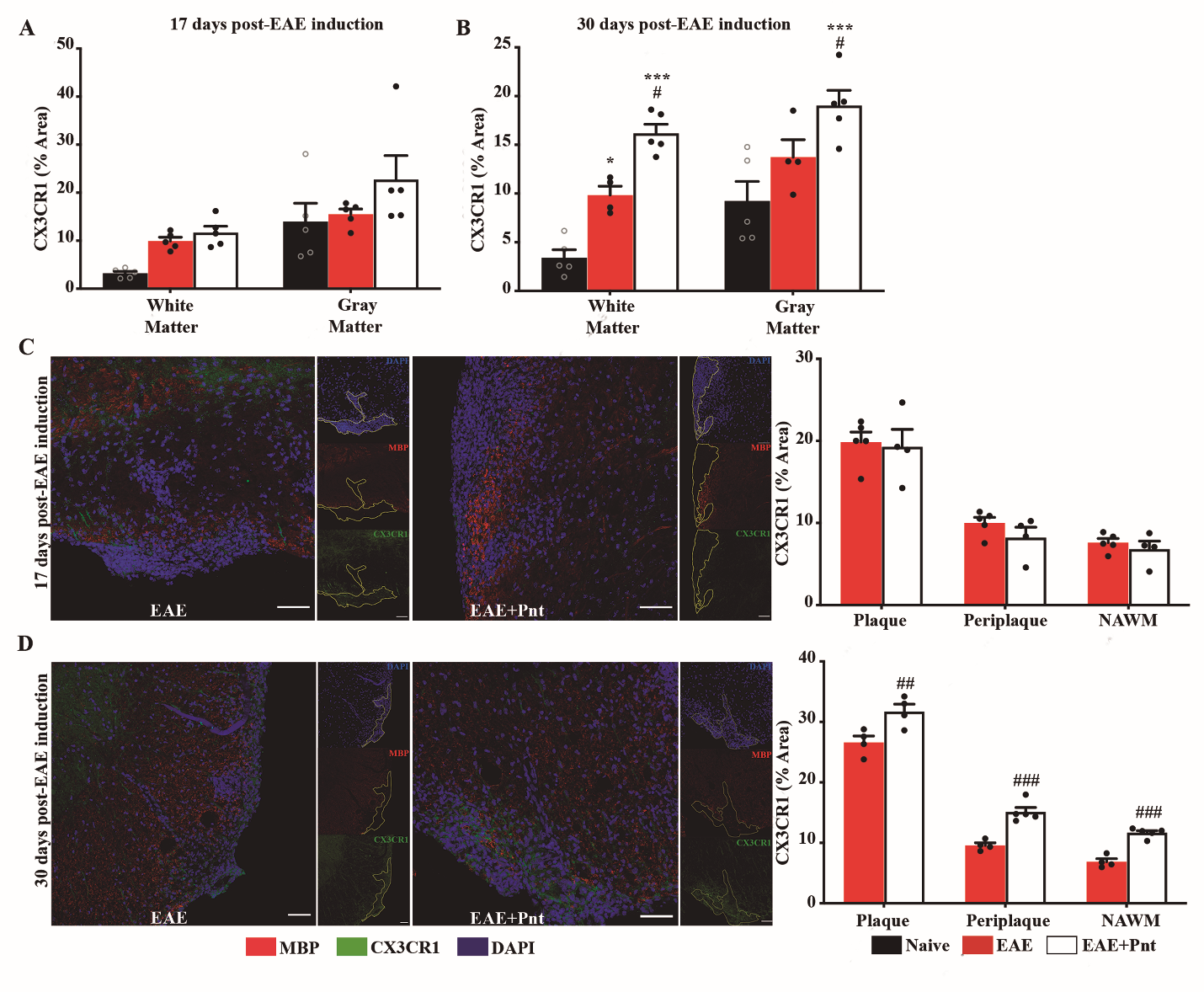


**Supplementary Figure 5** - **Pentamidine treatment promotes a CX3CR1-producing cells at chronic stages.** Graph bars representing the percentage of area stained for fractalkine receptor (CX3CR1) at **(A)** 17 and **(B)** 30 days post-EAE induction (dpi). Representative images immunostained for CX3CR1 (green) of the three delineated regions: plaque (P), periplaque (PP) and Normal Appearing White Matter (NAWM) and respective quantification at **(C)** 17 and **(D)** 30 dpi. Scale bar: 50 µm. Magnification: x40. One- and two-way ANOVA with Tukeys multiple comparisons was used for statistical significance (**P<*0.05, and ****P<*0.001 vs naïve; #*P<*0.05, ##*P<*0.01, and ###*P<*0.001 vs EAE) with n = 5 animals per group.


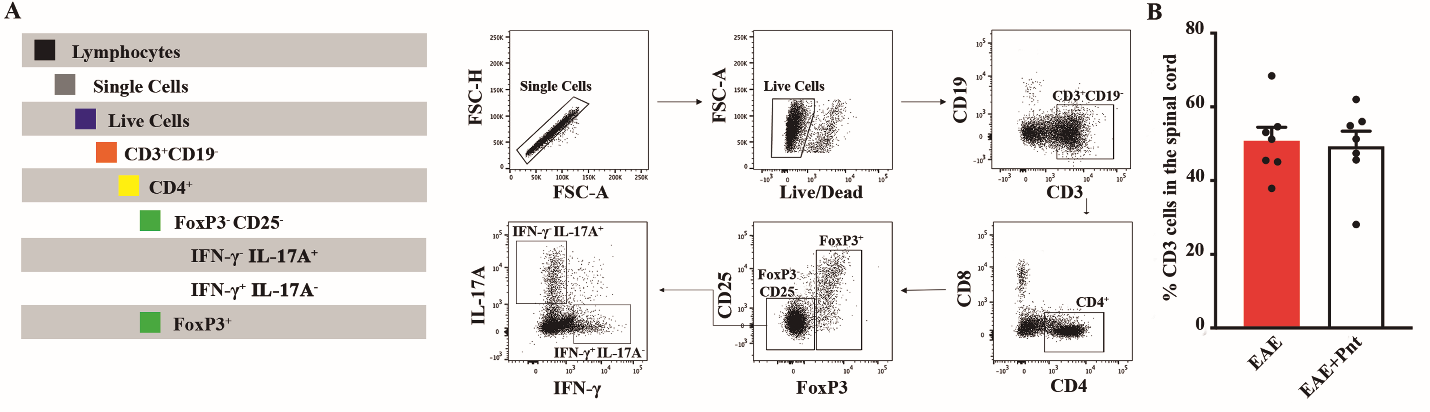


**Supplementary Figure 6 -** **(A)**T cell gating strategy. **(B)** Percentage of positive cells for CD3 population in the spinal cord with n = 7 animals per group.
